# A TinyMLOps Pipeline for Coarse-Grained Plant Disease Classification in Precision Agriculture

**DOI:** 10.3390/s26165029

**Published:** 2026-08-07

**Authors:** Hossein Aqasizade, Mattia Antonini, Massimo Vecchio, Fabio Antonelli

**Affiliations:** 1 Fondazione Bruno Kessler, 38123 Trento, Italy; haqasizade@fbk.eu (H.A.); m.antonini@fbk.eu (M.A.); fantonelli@fbk.eu (F.A.); 2 Department of Information Engineering and Computer Science, University of Trento, 38123 Trento, Italy

**Keywords:** deep learning, convolutional neural networks, edge AI, TinyML, TinyMLOps, plant disease detection, plant disease classification, smart agriculture, precision agriculture

## Abstract

Identifying plant health conditions is an emerging precision-agriculture and food-security challenge, intensified by deploying deep-learning models on memory- and power-constrained edge devices. We present a TinyMLOps pipeline spanning model design, optimization, quantization, and deployment across diverse edge devices, evaluated under controlled laboratory conditions. Using a dataset derived from the PlantVillage benchmark, 39 fine-grained classes are aggregated into three superclasses: healthy leaf, unhealthy leaf, and no leaves. The resulting system therefore performs plant health-status classification and background filtering rather than diagnosing specific diseases. We train a MobileNet-based convolutional neural network jointly optimized for classification accuracy and computational efficiency, adopting state-of-the-art hyperparameter optimization (HPO) tools. Five models are selected, four from the Pareto Front and one as the biggest evaluated model during HPO, converted to LiteRT and ONNX, and evaluated at float32 and post-training int8 precision on a Raspberry Pi Zero 2 W and an STM32H743ZI microcontroller. At float32, LiteRT is 1.87–2.65× faster than ONNX Runtime on the Raspberry Pi across all five models. Relative to their float32 LiteRT counterparts, the int8 LiteRT models are 2.83–3.67× smaller on disk and 21.3–31.2% faster on the same board, at a cost in F1-score of between 0.0010 and 0.0068. On the microcontroller, only the two smallest models deploy at both precisions; for these, the fully quantized int8 variants are 4.3× faster and 3.85× smaller in MCU flash footprint than the float32 counterparts. The mid-range model fits the 2 MB flash and 1 MB RAM budget only when quantized, while the two largest models exceed it in every configuration tested.

## 1. Introduction

As the need for food security grows, precision farming stands as a powerful ally, leveraging the most cutting-edge technologies to enhance crop production. Every year, significant portions of farm production are wasted due to recurring pests, devastating diseases, harsh weather patterns, and subtle soil erosion, all of which contribute to these losses. The precision farming paradigm, enabled by modern technology to make farming more efficient and productive, provides a powerful answer to these growing threats. To protect plants and secure the farming economy, the ability to make early, accurate plant disease identification remains fundamental, as it enables quick action before a widespread epidemic. Traditionally, this function was provided by biological methods, as in the case of “sentinel” plants [1], which exhibit symptoms indicative of particular pathogens, serving as a sort of natural early warning system. Although theoretically sound, traditional methods are bogged down by practical constraints. They are quite time- and labor-intensive to plant, grow, and continuously monitor. Further, human visual inspection is prone to error, particularly in misinterpreting or completely overlooking disease symptoms [2]. As a countermeasure to the limitations of manual, biology-focused monitoring, the farming industry continues to embrace technological innovations. Digital farming leverages solutions such as computer vision, Internet of Things (IoT) sensor systems, and AI-driven predictive analytics to provide faster, scalable, and more reliable real-time monitoring of crop health [3]. Among the array of methods, the combination of computer vision and deep learning has proven one of the most effective for mechanizing plant disease classification from images [4].

The noteworthy aspect in this case is the direct execution on edge devices, from microcontrollers (MCUs) and single-board computers (SBCs) to real-time, in-situ processing of a continuous stream. Such embedded settings are themselves defined by tight computational constraints that profoundly impact model design, from architectural size (megabytes vs. kilobytes) to data types (float32 vs. int8), among others. In real agricultural deployments, connectivity is often intermittent or entirely unavailable, and power budgets are limited. For this reason, disease detection is better performed locally, directly on embedded devices, enabling real-time responses while minimizing communication, energy consumption, and operational cost. Systematically managing these restrictions is necessary, and the TinyMLOps approach provides a systematic methodology for model design, optimization, and execution on devices with limited capabilities [5].

This work describes the development of a computer vision system based on TinyMLOps principles, designed to run an optimized deep neural network model on embedded platforms for plant disease monitoring. The system uses the state-of-the-art MobileNetV1 backbone architecture [6] as a pre-trained feature extractor, while a custom final classification layer is trained from scratch. Optuna [7] is utilized for hyperparameter tuning, balancing detection accuracy and model complexity.

**Figure 1:**
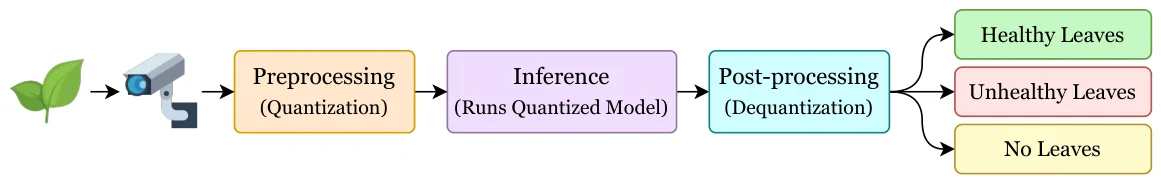
Envisioned on-device inference pipeline for real-time plant-health screening under field conditions. A captured image is preprocessed and passed to the quantized model, which classifies the input as a healthy leaf, an unhealthy leaf, or no leaf.

The experiments were conducted on the PlantVillage dataset, a widely used benchmark in the plant disease detection literature, consisting of high-quality leaf images acquired under controlled laboratory conditions. While this dataset supports rigorous and reproducible evaluation of the vision-sensing pipeline, it does not reflect the full complexity of outdoor deployments, including variable lighting, occlusions, and background clutter. The quantitative results reported here should therefore be interpreted as benchmark performance. To assess operability beyond PlantVillage, the system is additionally evaluated qualitatively on previously unseen, locally collected leaves (Section 4.2.3). This work contributes the systematic integration of model optimization, deployment, and hardware evaluation into a reproducible TinyMLOps lifecycle for heterogeneous edge devices in precision agriculture. The ultimate operational objective is real-time, on-device plant-health screening under field conditions. The envisioned inference pipeline for field deployment is illustrated in Figure 1.

The main contributions of this article are summarized as follows:

1. Designed and implemented an end-to-end TinyMLOps pipeline for edge deployment, integrating state-of-the-art lightweight CNN architectures (e.g., MobileNet) and covering the full workflow from model development to on-device inference;
2. Comprehensive hardware-level benchmarking and deployment validation on heterogeneous edge devices, namely a Raspberry Pi Zero 2 W and an STM32H743ZI microcontroller, providing detailed analysis of inference latency, memory footprint, and accuracy trade-offs, and establishing the feasibility of on-device inference.
3. Optimization and adaptation of plant disease classification models for multiple resource-constrained devices.

The remaining parts of the paper are organized as follows: Section 2 provides an overview of research in Edge AI and TinyMLOps, reviewing prior frameworks, deployments, and optimization approaches. Section 3 details the proposed workflow, including dataset preparation, model design/training, optimization, and edge-device deployment and configuration. Section 4 reports the experimental results on both target platforms, together with a discussion of the observed trade-offs, limitations, and a roadmap towards field deployment. Finally, Section 5 concludes the paper by summarizing the main findings and outlining directions for future work.

## 2. Related Work

Research related to this study spans multiple intersecting areas, including TinyMLOps frameworks, edge AI architectures, model and optimizations for TinyML, and plant disease detection on edge devices. This section reviews the most relevant contributions across these dimensions and positions the proposed work within the existing literature.

### 2.1. Vision-Based Plant Disease Detection in Precision Agriculture

Wadhwa et al. [8] propose a generalized hybrid CNN ensemble for multi-crop disease classification that uses F1-score driven Dirichlet weighting and CatBoost stacking, with SHAP-based explainability, while Sakthipriya et al. [9] propose a precision agriculture approach for rice nutrient management that uses genetic algorithms to automatically design and optimize CNN architectures. Their GENCNN framework focuses on model automation and accuracy, aiming to improve nitrogen management and reduce fertilizer costs, but does not address deployment, edge constraints, or operational lifecycle aspects. Sivakumar et al. [10] propose a computer vision–based crop and weed segmentation framework using federated ensemble learning across heterogeneous multi-crop field datasets. Their approach enables scalable, privacy-preserving model training and incremental deployment without centralized data sharing. Khan et al. [11] propose a MobileNetV3-Small-based model for plant disease classification on edge devices. Their approach focuses on achieving high accuracy on the PlantVillage dataset and applies post-training quantization to reduce model size. Several additional studies provide relevant accuracy–complexity references on PlantVillage, including classifiers based on AlexNet and GoogLeNet [12], InceptionV3 [13], a compact nine-layer CNN [14], and a modified InceptionV3 [15]. Their reported predictive metrics and parameter counts are interpreted in Section 4.4, with explicit consideration of differences in class granularity and experimental protocol. Arratia et al. [16] propose the BODOQUE (Bimodal Observational Device for Optimizing Quantification of Ephemeral streams), a systematic framework for deploying TinyML in real-time environmental monitoring that combines low-power sensing with event-triggered, high-performance processing to minimize energy consumption. Other agricultural applications emphasize model design rather than operational deployment. In [17], the authors introduced a general inference pipeline for plant disease detection running on a small single-board computer (i.e., a Raspberry Pi Zero 2 W). Building on that foundation, this paper focuses on the specific optimizations required for efficient on-device execution at the network edge. Although these studies demonstrate the effectiveness of deep learning for agricultural disease monitoring, many prioritize classification accuracy over deployment feasibility, runtime efficiency, and operational lifecycle management on embedded sensing platforms.

### 2.2. Lightweight Deep Learning Models for Edge Agricultural Monitoring

To go beyond hardware limits, scientists have attempted architectural and algorithmic advancements. Larian et al. [18] introduced the InTec framework and experimented with a three-layer architecture. They found that dividing the ML pipeline across the thing, edge, and cloud layers might significantly reduce latency and network traffic. In a more comprehensive study, Jenhani et al. [19] conducted an experimental investigation of split learning. On a customized testbed composed of ESP32 boards, they experimented with the efficiency of a partitioned MobileNetV2 model across different low-power wireless protocols. They identified optimal split points to balance on-device computation and communication overhead. Rather than disseminating the model, Hayajneh et al. [20] examined the potential to enhance the model’s adaptability. Their study on on-device transfer learning demonstrated its feasibility for enabling models to acquire knowledge from novel data directly at the edge. Cerioli et al. [21] expressly stated the advantage of strongly specialized optimization in their research on the detection of microplastics. They built a tailored compiler to generate native C code for their model without relying on standard inference engines. From their experimental data, they found that their technique achieved significant increases in latency, memory use, and energy efficiency compared with typical techniques. Similarly, Lê et al. [22] are interested in the run-time dynamics of TinyML and introduce a new TinyMLOps approach to real-time frame-based event classification on ultra-low-power microcontrollers. They presented a comparison using available frameworks, such as LiteRT and NNoM, to support their strategy. They even use TinyML to secure consumer IoT devices.

### 2.3. TinyMLOps and Edge AI Frameworks for Resource-Constrained Deployment

The Edge AI paradigm is a revolutionary shift that is driving new approaches to deploying applications on resource-limited devices, especially in verticals such as precision agriculture. The peculiar restrictions on these systems have led to the definition of Tiny Machine Learning Operations (TinyMLOps) [5]. Antonini et al. research [5] presents a comprehensive, end-to-end industrial IoT application for operationalizing anomaly detection in submersible water pumps. Additionally, this paper utilizes readily available, inexpensive hardware and presents a rigorous empirical study to support its findings. Moreover, they provide a formal, detailed description of the conceptual TinyMLOps framework, which applies classical machine learning orchestration principles to the unique domain of non-Linux-based, far-edge devices. However, Leroux et al. [23] say wide deployment is impeded by operational obstacles that transcend computation and reach to model observation, management, security, and integrity verification on distributed device populations. To address these operational concerns, various MLOps frameworks have been developed.

For an open-source alternative, Peltonen et al. suggested LinkEdge [24], an end-to-end toolset for connecting edge devices and supporting MLOps. They tested the system by creating a model to predict air-pressure failures in trucks and concluded that their open-source alternative performed as well as commercial tools while providing freedom from vendor lock-in. Extending on-device execution boundaries, Min et al. [25] created a multi-tenant sensory edge device runtime called SensiX++. They discovered that a resource-aware model server and an adaptive scheduler could be adopted to securely and efficiently run multiple isolated models on a single device. To overcome the practical hurdles across the entire workflow, Huang et al. [26] proposed the RIOT-ML toolkit. They propose a “batteries-included” package that includes a low-power OS, a model transpiler, and a secure over-the-air (OTA) update system and demonstrate its usefulness for benchmarking model performance on diverse IoT hardware. In other contributions, Karras et al. [27] contend that effective data management is a condition for successful edge AI. Towards that end, they designed and delivered a suite of TinyML-specific data-cleaning, clustering, and compression algorithms.

In other work, Katib et al. [28] designed a Deep Learning technique to secure IoT consumer devices with TinyML Driven Real-time Anomaly Detection for Predictive Maintenance (DLTML-RTADPM), to achieve real-time anomaly detection and predictive maintenance. Their approach comprises Z-score normalization as the data preprocessing method, the Fennec Fox Optimization Algorithm to select features, a bidirectional long short-term memory (BiLSTM) network for anomaly detection, and the Jaya optimization algorithm to optimize hyperparameters. Flores et al. [29] suggest a system of tiny machine learning tasks under the Internet of Intelligent Vehicles. Two ESP32 units are used in their solution, with one serving as a radio station and the other as an onboard diagnostic scanner. These updated models are then sent over a wireless channel. This system shows a marked improvement in model efficacy post-update. Automated plant disease detection has attracted significant research interest, with numerous studies leveraging deep learning and computer vision for image-based diagnosis in precision agriculture [30,31,32,33]. However, most works prioritize classification accuracy over deployment feasibility.

### 2.4. Summary and Research Gaps

The novelty of this work lies in operationalizing TinyMLOps principles for precision agriculture, specifically through a quantized, multi-objective-optimized MobileNetV1 pipeline for plant disease classification on resource-constrained devices. By leveraging heterogeneous edge hardware (Raspberry Pi Zero 2 W and STM32H743ZI), runtime frameworks (LiteRT, ONNX), and experiment tracking tools (MLflow), an end-to-end TinyMLOps pipeline is proposed that is both technically robust and cost-effective, enabling scalable deployment in agricultural applications. Unlike prior works that focused primarily on conceptual frameworks or partial workflows, this study presents a TinyMLOps lifecycle, covering model design and hyperparameter optimization with Optuna, mixed-precision quantization, deployment, and live monitoring on heterogeneous edge devices. The resulting workflow provides an experimentally validated example of applying TinyMLOps in agricultural edge AI scenarios. The differences between this work and existing approaches are summarized in Table 1 for clarity.

## 3. System Design Overview

**Figure 2:**
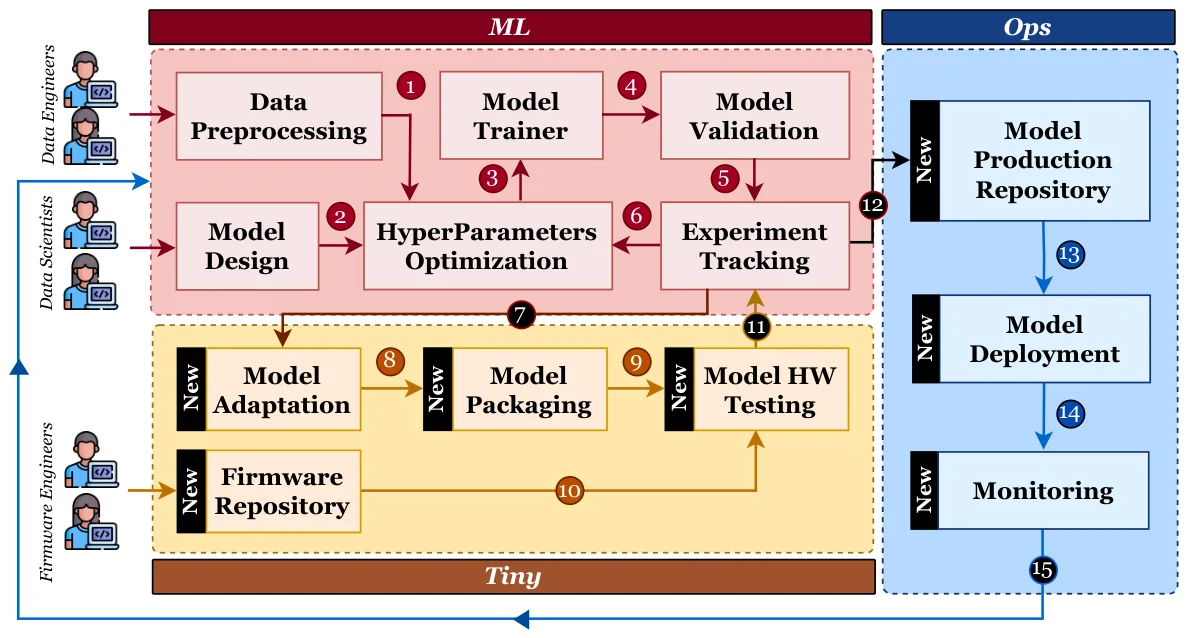
A TinyMLOps Workflow; an end-to-end pipeline that integrates three domains: Machine Learning (ML) for model development, Tiny for hardware-specific adaptation, and Operations (Ops) for deployment and monitoring. The ‘New’ markers are the core focus of this work w.r.t. [17].

This section presents the TinyMLOps workflow adopted to design, implement, and evaluate the feasibility of a vision-based plant disease classification pipeline, from dataset preparation through model design, training, optimization, conversion, deployment, and monitoring. Figure 2 details each step from data preprocessing and model design in the Machine Learning (ML) phase, through model conversion and testing in the Tiny phase, to final deployment and monitoring in the Operations (Ops) phase.

### 3.1. ML Pipeline: Model Creation & Optimization

The first stage is to learn a high-quality, precise classification model. This is done through data preprocessing, defining the model architecture, and using a multi-objective optimization process to achieve an optimal performance-size trade-off. The ML process was run on a MacBook Pro with an M3 Pro chip, a 12-core processor, and 18 GB of memory, which provided sufficient computing power to handle large computations.

#### 3.1.1. Step 1 & 2: Data Preprocessing & Model Design

**Figure 3:**
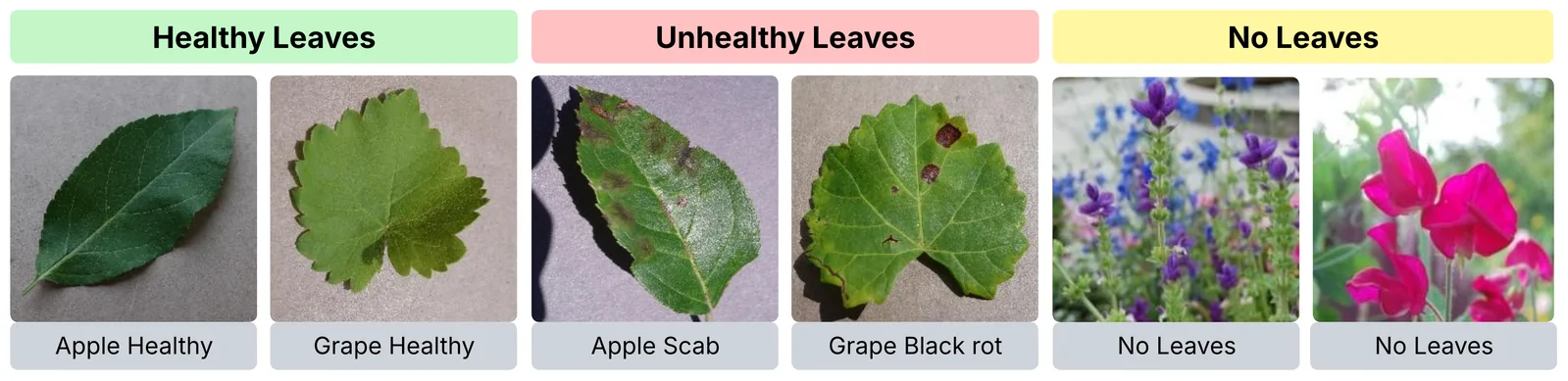
Examples of Healthy, Unhealthy, and No Leaves images extracted from PlantVillage [14].

The large PlantVillage dataset was utilized [14], consisting of 61,486 images classified into 39 different classes: 12 healthy plant leaf sample varieties, 26 classes representing different leaf disease occurrences, and a separate class of no-leaf image samples (e.g., flowers, houses, animals). A visual representation of some of the samples can be seen in Figure 3, and the class distribution is given in Table 2.

The 39 individual classes were aggregated into three superclasses: healthy leaves, unhealthy leaves, and no leaves. This coarse-grained formulation preserves the screening goal of distinguishing healthy from diseased plants while rejecting non-leaf inputs.

The initial image distribution showed a large imbalance: 43,875 as unhealthy, 16,468 as healthy, and only 1143 as no leaves and this poses a risk of producing a biased model that unduly privileges the majority class (unhealthy) at the expense of minority classes, especially the no leaves class.

To minimize class imbalance, data augmentation strategies were applied using the Albumentations library [34] and following an augmentation-based approach similar to that described in [35]. The augmentations involved a sequence of geometric and photometric transforms, such as horizontal and vertical flips and random rotations up to 25 degrees. Brightness and contrast adjustments were also used to bring in changes in lighting conditions. Gaussian blur and Gaussian noise were also used to simulate textural variation and enhance the model’s robustness. As a result, the no leaves instances thus increased from 1143 to 15,325, resulting in a better-balanced dataset.

In addition, all images were resized to an identical dimension of 128 × 128 pixels to meet the MobileNet [6] architecture’s input size requirement and to maintain homogeneity across the entire dataset to facilitate model generalization. The preprocessed and balanced dataset created is available in the public domain at AgrifoodTEF Data Space [36]. Next, the complete dataset was split into training, validation, and test sets at 60/20/20, respectively. The division ensures that a substantial portion of the data is used for model training.

Following data preparation, the next step (Step 2) is to create and train the classification model. The problem is addressed through transfer learning, which enables the reuse of pre-trained visual representations while reducing training cost and data requirements. In this setting, the convolutional backbone acts as a feature extractor, while only a task-specific classification head is trained for the target plant-disease classification task.

Given the deployment constraints of heterogeneous edge devices, the backbone must provide a suitable trade-off between accuracy, computational cost, memory footprint, and toolchain compatibility. Several compact CNN architectures are commonly considered for edge inference, including EfficientNet-Lite, ShuffleNet, SqueezeNet, compact ResNet variants, and the MobileNet family. Among these, MobileNet is particularly attractive for constrained devices because its depthwise separable convolutions substantially reduce the number of operations and parameters.

In this work, MobileNetV1 is adopted as the backbone. Although more recent MobileNet variants, such as V2 and V3, introduce architectural improvements aimed at improving accuracy–efficiency trade-offs, they also rely on more complex building blocks that may reduce operator-level compatibility and deployment robustness on constrained MCU targets. MobileNetV1 was therefore preferred for its simpler architecture, reduced computational footprint, and compatibility with the STM X-CUBE-AI toolchain used for MCU deployment. Its capacity is controlled by the α width multiplier, which scales the network to match the resource constraints of the target edge device.

A task-specific classification head is added on top of the frozen MobileNetV1 backbone. This head consists of a fully connected layer with *N* units, followed by a Dropout layer with rate Dr, and a final output layer mapping the extracted features to the three target superclasses. The values of α, *N*, and Dr are treated as tunable hyperparameters and optimized through the procedure described below to balance classification performance and computational cost. Since only the classification head is updated during training, the number of trainable parameters is substantially reduced, limiting training cost and helping mitigate overfitting.

#### 3.1.2. Step 3 & 4: Hyperparameter Optimization & Model Training

Given the deployment target of low-resource platforms, including SBCs and MCUs, hyperparameter tuning was formulated as a multi-objective optimization problem balancing F1-score maximization and model-size minimization. The F1-score was used to account for class imbalance, whereas the total number of parameters served as a hardware-agnostic proxy for model complexity. The tunable hyperparameter space Θ is reported in Table 3. Although several hyperparameters were optimized, the width multiplier was emphasized in the analysis because of its direct impact on model size, memory footprint, and edge deployability.

(1) Θ={α,Dr,Bs,N}.

To frame it as a minimization problem, with respect to both the objective functions, with optimal solution converging to the origin (0,0), the first objective was rewritten as 1−F1score(Θ) and the second objective function was simply the number of parameters, num_params(Θ). Writing them in this manner ensures that small values indicate better performance. The strict multi-objective function to be minimized was therefore written as:

(2) $$\min_{\Theta }\left(1-F1\;\mathrm{score}(\Theta ),\;\mathrm{num}\_\mathrm{params}(\Theta )\right)$$

Given the computationally expensive nature of the optimization problem, conducting an exhaustive search of the hyperparameter space was infeasible due to the vast number of possible combinations. Therefore, a metaheuristic search strategy was employed to efficiently explore the solution space and identify (sub-)optimal solutions belonging to the Pareto front, a set of non-dominated solutions that represent optimal trade-offs, where improvement in one objective function necessarily entails a compromise in another [37].

This optimization process is particularly crucial for real-time edge applications where computation and energy budgets are limited but predictive reliability must remain high. Training employed standard optimization techniques, such as the Adam optimizer and early stopping, while tuning hyperparameters like dropout rate, batch size, and layer width to maximize efficiency. Adam optimizer was selected for its adaptive learning rate, with an initial learning rate of 0.0001.

Although the dropout search space allowed values up to 0.9, high-dropout configurations were not retained in the final Pareto front. As reported in Table 4, all five selected Pareto-optimal models use dropout rates of 0.5 or lower, suggesting that NSGA-II favored moderate regularization under the joint objectives of maximizing F1-score and minimizing model size.

The batch size Bs was also optimized as a hyperparameter to find an optimal trade-off between training stability and resource usage. To ensure deterministic repeatability of our experiments, the pseudo-random number generator was seeded with the fixed value 73 before initiating the training protocol. Moreover, for multi-objective optimization, the Optuna v4.3.0 library [7] and its NSGA-II implementation [38] were used, both of which are tested and functioning multi-objective genetic algorithms. The hyperparameter set of Table 3 was utilized as an integer-based chromosome structure for the number n=4 genes, where the value of each gene was encoded to the index of the respective hyperparameter choice (for instance, α=0.5 was encoded to index 1). The NSGA-II algorithm was executed with a population of 64 members. The solution selection used a tournament selection with elitism based on rank and crowding distance. For mating between parent solutions, a uniform crossover is employed with a crossover probability of 0.9 and a gene exchange probability of 0.5. Mutation occurs at the gene level, with each gene independently altered with probability 1/n=1/4 to maintain genetic diversity. The crossover and mutation operations are applied sequentially and independently to the offspring, fostering diversity and improving search-space exploration.

At each optimization trial (Step 3), NSGA-II samples a candidate hyperparameter configuration from the search space reported in Table 3 (Step 4). The corresponding model is instantiated, trained, and evaluated in terms of F1-score and total parameter count, enabling the optimizer to identify accuracy–complexity trade-offs without exhaustively exploring the full design space.

The optimization was implemented with Optuna and run for 20 NSGA-II generations, resulting in 1280 evaluated candidate models. This budget was selected to provide sufficient search diversity while keeping the computational cost of repeated model training tractable. The Pareto-optimal models were subsequently exported to ONNX and LiteRT formats, including a quantized LiteRT version. For each exported model, the impact of conversion and quantization on classification performance was then assessed.

#### 3.1.3. Step 5 & 6: Model Validation & Experiment Tracking

During the hyperparameter optimization phase, each trained model resulting from a unique hyperparameter configuration (often referred to as a trial) was evaluated using the same validation set (Step 5). To obtain the hyperparameter search, an end-to-end computing infrastructure was set up to run experiments and report their results. The software stack for this consisted of TensorFlow v2.19.0, which served as the hub of our software package and was used to develop, train, and optimize deep learning models, along with Python v3.12.8. To manage experiments, a containerized version of MLflow [39] (version 2.18.0) was run on a CloudLab [40] testbed node. CloudLab was selected due to its prior experimental validation [41,42] as being appropriate and adaptable for scientific experiments. Everything related to experimental results, hyperparameters, F1 scores, learned model parameters, and training logs was properly captured using an MLflow server (Step 6). Tracking was done centrally and aids reproducibility while providing a clear representation of the Pareto front of non-dominated solutions (i.e., solutions for which no other solution is better in all objectives [43]), to select candidate models at the subsequent stage.

### 3.2. Edge Device Adaptation with Hardware-Specific Optimization

Once an optimal set of models was identified, work was done to adapt and prepare them to run on resource-constrained hardware. This involves several operations, including conversion, quantization, and running through a test device.

#### 3.2.1. Steps 7 & 8: Model Adaptation & Packaging

After training, there is a pool of models that lie on the Pareto front and make trade-offs among the objective functions. From this Pareto front, five optimal solutions (four Non-dominated and one Dominated) were selected along with their corresponding models, which is identified as the best in some sense: model A has the lowest number of parameters among all the solutions over the front, while models B (Global Pareto Front Knee), C and D are the trade-offs between the model size and the detection performance metric for α=0.25, α=0.5, and α=0.75, respectively. Additionally, model E, the largest configuration evaluated during hyperparameter optimization (α=1.0), is included.

Each model was exported from Keras to two deployment formats to preserve flexibility on the target device:

- LiteRT (Lite Runtime) [44] is an open-source performance runtime developed by Google, formerly called TensorFlow Lite (TFLite), used for running TensorFlow models on devices. It supports rapid machine learning inference across smartphones, microcontrollers, and embedded Linux platforms. LiteRT is a low-latency, small-footprint, low-energy product, making it ideal for real-time execution on edge hardware.
- ONNX (Open Neural Network Exchange) [45] is an open-source format developed jointly by Microsoft and Facebook to improve interoperability among different machine learning frameworks. ONNX provides a standardized representation of machine learning models, enabling developers to train models in one framework and deploy them in another.

As part of Step 7 (adaptation) and Step 8 (packaging), quantization was applied to reduce model size and inference cost. Quantization is the process of lowering the numeric precision of model activations and weights, typically from 32-bit floating point (float32) to more space-saving representations like 16-bit floating point (float16) or 8-bit integer (int8). As a result, it minimizes model size, memory footprint, and inference latency, all of which are critical on resource-constrained hardware such as single-board computers or microcontrollers. There are various quantization methods; post-training quantization was chosen because it is straightforward to implement after model training, thus no model retraining is necessary. Post-training quantization can be classified into three main categories: dynamic-range, full-integer, and mixed-precision quantization. Dynamic range quantization reduces weight precision while keeping activations in floating-point representations, whereas full integer quantization converts both weights and activations to integer representations.

**Figure 4:**
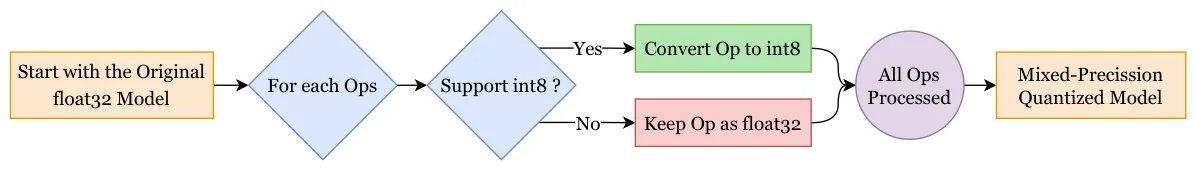
Mixed-Precision Quantization Workflow.

In this study, a mixed-precision approach, known as “float fallback” quantization, was adopted. As documented by Google AI Edge [46], this method quantizes the model’s weights and activations to int8. In contrast, the model’s input/output tensors and any unsupported ops remain in float32 for compatibility. Using this technique required building a representative dataset from a random subset of the preprocessed training images, enabling the model converter tool to effectively calibrate the quantized model. This approach achieves a balance among memory footprint, latency, and accuracy on resource-constrained hardware. The workflow for mixed-precision quantization is illustrated in Figure 4.

#### 3.2.2. Steps 9, 10 & 11: Model Hardware Testing & Feedback

At this stage, the process transitions from packaging trained models to verifying their performance on real hardware, closing the loop with feedback for iterative optimization. The objective is to ensure that each candidate model is both compatible with and efficient on the target embedded platforms and determine the runtime configuration and precision settings that best satisfy deployment constraints such as latency, memory usage, and accuracy. First, (Step 9) bridges model packaging with hardware evaluation, enabling models to be transferred to the target devices, specifically a Raspberry Pi and an STM-based microcontroller. Each device is initialized with a clean firmware image from the Firmware Repository, ensuring controlled, reproducible testing conditions (Step 10). The Raspberry Pi is initialized with a Raspberry Pi OS Lite (64-bit, Debian Bookworm), while the STM microcontroller is loaded with a validation firmware available in the STM X-CUBE-AI (https://www.st.com/en/embedded-software/x-cube-ai.html, accessed on 1 July 2026) toolchain. Once the software firmware integration required for on-device testing is complete, the packaged models are executed and evaluated using the firmware deployed on each device. The firmware provides a complete runtime environment for evaluating, collecting, and archiving model performance. Models packaged in both LiteRT and ONNX formats were compared, using identical inputs, to evaluate inference latency, memory footprint, and compatibility.

Next, within each framework, both float32 and quantized (int8/mixed-precision) variants were tested to quantify trade-offs between computational speed, memory footprint, and accuracy. To emulate resource-constrained edge-execution conditions, all evaluations were conducted under single-thread/core execution, reflecting typical edge-device limitations. For ultimate performance verification, chosen models were executed and benchmarked on Raspberry Pi Zero 2 W running Raspberry Pi OS Lite (64-bit, Debian Bookworm). The entire setup provided us with a robust, efficient platform for our deep learning tests. Each benchmark run included a warm-up phase followed by multiple inference iterations, 1000 runs on the Raspberry Pi and 10 runs on the STM microcontroller, to reach a stable steady state. System-level metrics such as inference latency, resident set size (RSS) memory, and swap or equivalent memory utilization were gathered using device-specific monitoring programmatic interfaces: psutil and /proc on Raspberry Pi, stedgeai-core (https://www.st.com/en/development-tools/stedgeai-core.html, accessed on 1 July 2026) toolkit on the STM microcontroller. These experiments relate to the *Verify* steps of the TinyMLOps cycle [5].

Outputs were written to CSV files and later gathered across runs for analysis. The average, minimum, maximum, and standard deviation of inference time, average memory usage, and model file size were also collected on an MLflow server in CloudLab. Model artifacts and result logs were also pushed to the MLflow server (Step 11), enabling centralized experiment tracking, reproducibility, and integration with the TinyMLOps lifecycle. This feedback loop helped us compare the on-device runtime performance of each model variant and pick the most efficient variant for production deployment. To prevent runs from interfering with one another, a two-minute pause was added between runs, allowing system resources to stabilize before the next inference. Using this approach, runtime performance, memory usage, and overall feasibility of all model variants were thoroughly evaluated under typical edge deployment conditions.

### 3.3. Ops: Production Deployment & Monitoring

Following exhaustive benchmarking and validation, the final step in the TinyMLOps workflow is operationalizing the selected models. The workflow ensures that the tested and fine-tuned models are properly orchestrated, deployed, and monitored in a production environment, thereby closing the end-to-end lifecycle from development through real-world deployment.

#### 3.3.1. Steps 12 & 13: Model Production Repository & Deployment

Based on hardware testing results, quantized LiteRT models consistently achieved superior inference speed and lower memory usage. These tested models, along with their performance logs and metadata, were further promoted to the Model Production Repository (Step 12). The repository is the sole source of truth for production-ready artifacts with versioning and reproducible deployment. These included the lightweight, quantized LiteRT models, which were found to be most efficient in terms of inference speed and memory consumption on the hardware under test. Saving the models as production-ready artifacts, along with associated metadata and MLflow performance logs, provides versioning and enables reproducible deployments. Then, the selected models from the repository are deployed on edge devices (e.g., Raspberry Pi Zero 2 W and STM32 microcontroller) (Step 13). This process is automated, enabling scalable, consistent deployments of new model iterations. The deployment method leverages the efficient LiteRT, selected for its low latency and low resource requirements, making it well-suited to the constraints of the targeted hardware.

#### 3.3.2. Steps 14 & 15: Live Monitoring & Continuous Improvement

The lifecycle concludes with a monitoring stage (Step 14) to observe model and system behavior after deployment. Runtime indicators such as prediction confidence, output entropy, inference latency, memory usage, and hardware resource utilization can be collected to characterize both predictive behavior and execution efficiency in the target environment. This deployment-time observability complements offline benchmarking by enabling the detection of changes in operating conditions, input data characteristics, or resource usage patterns.

The resulting information supports the feedback stage (Step 15) of the TinyMLOps lifecycle. When relevant deviations from the expected behavior are observed, monitoring data can guide subsequent inspection, dataset update, and retraining activities. In this work, these mechanisms are defined as part of the proposed lifecycle architecture; their long-term validation in open-field deployments is considered future work.

## 4. Experimental Results

This section presents the results of our experiments conducted on two representative edge platforms: a single-board computer (Raspberry Pi Zero 2 W with 512 MB of RAM) and a widely used microcontroller (STM32H743ZI with 1 MB of RAM and 2 MB of flash memory). First, the best models balancing size and accuracy were identified. Subsequently, the execution performance of the models on the Raspberry Pi Zero 2 W board across different runtime frameworks and quantization settings was evaluated, including an analysis of performance gains and model accuracy losses. Finally, the models’ deployability was assessed on the STM32H743ZI microcontroller to validate their feasibility and performance under strict memory and computational constraints.

### 4.1. Model Selection Trade-Off

**Figure 5:**
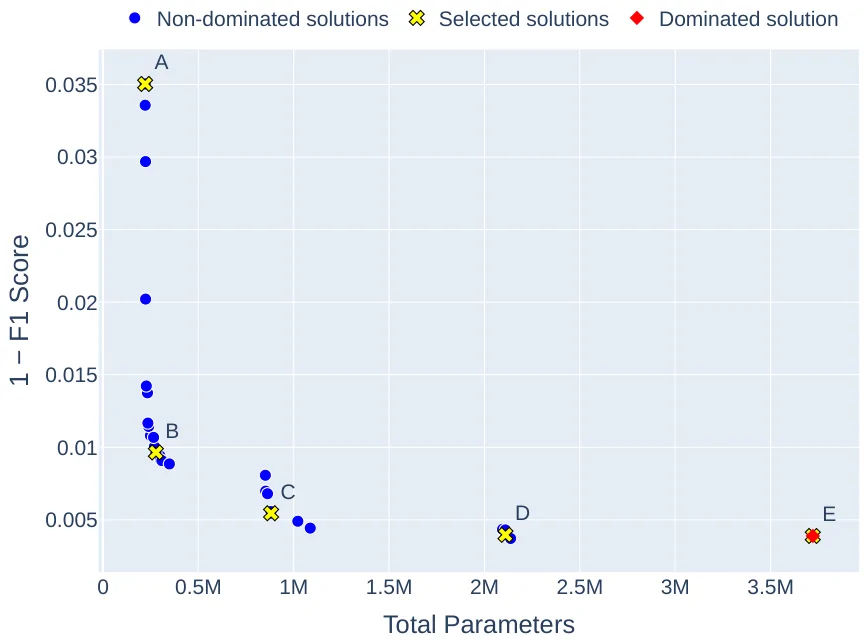
Pareto front with the optimal solutions after 20 generations, marked as dots, of 1−F1score versus total parameters. Cross markers mark selected models for various α values. Finally, the diamond marker marks a dominated solution with the highest number of parameters.

A metaheuristic search between model size and model performance 1−F1score using the NSGA-II algorithm was conducted, resulting in the Pareto front approximation illustrated in Figure 5, where the x-axis represents the total model parameters (model size), while the y-axis 1−F1score reflects classifier performance. After 20 generations, the metaheuristic optimization process identified 28 solutions lying on the Pareto front. Based on these findings, five representative models were selected (as discussed in Section 3.2.1) to cover the entire range of α. Table 4 presents the selected models A–E (four non-dominated and one dominated), which are marked as crosses on the front.

To contextualize the trade-off, Table 5 compares the five selected Keras float32 models against representative state-of-the-art CNN classifiers trained on PlantVillage. Our proposed configurations span 0.221–3.72 M parameters, with F1 scores ranging from 0.965 to 0.996. Model A is the smallest configuration at 0.221 M parameters, while Model B achieves an F1 score of 0.990 with 0.277 M parameters. Model C achieves an F1 score of 0.994 with 0.881 M parameters, closely matching the 0.9 M-parameter MobileNetV3-Small baseline reported by Khan et al. [11], which achieves an F1 score of 0.995. Note that these baselines address fine-grained classification, so the comparison concerns parameter efficiency rather than diagnostic performance.

#### Search-Space Coverage and Evaluation Costs

Table 3 shows the hyperparameter space, which is finite and discrete, totaling α(4)×Dr(6)×Bs(4)×N(505)=48,480 configurations. In our study, NSGA-II reached the reported front by evaluating 1013 unique configurations (2.09% of the space; 267 of the 1280 evaluations were repeats). Because each selected model A–E is an enumerable grid point, an exhaustive sweep would evaluate those same configurations. The achievable Pareto front over the discrete grid is thus determined by the objective landscape rather than by the search strategy, which governs only which points are evaluated and at what cost, not the front itself. The metaheuristic therefore approximates the relevant Pareto-optimal region using substantially fewer evaluations (lower cost) than an exhaustive grid search. Matching the grid resolution would require ∼48× more unique evaluations overall.

Moreover, an exhaustive grid search is *order-dependent*: the trial at which it first reaches a given model equals that model’s rank in the sweep, set purely by loop order and direction (ascending ↑ or descending ↓). For a deployment-oriented evaluation, we fix one representative traversal: ascending order with α as the outermost hyperparameter, followed by Dr, Bs, and *N*, where *N* varies fastest and α varies slowest. Under this ordering, models A and B (α=0.25) can be reached at trials 7072 and 5774, respectively, whereas model C (α=0.5), located near the front knee, can be reached only at trial 15,243, after the entire α=0.25 block has been evaluated. Models D (α=0.75) and E (α=1.0) require 25,602 and 40,367 trials, respectively.

Considering average training times of 13.8, 14.0, 18.2, and 25.0 min for α = 0.25, 0.5, 0.75 and 1.0, respectively, with each model-training run executed using a single CPU thread, the time required to discover models A–E ranges from approximately 55 days for model B (which has a smaller Dr than model A) to approximately 456 days for model E, while the entire grid exploration would require approximately 597 days. Table 6 summarizes the trial at which each model can be discovered and the corresponding estimated cumulative computation time. In contrast, our metaheuristic approach required approximately 15 days to evaluate 1280 trials and identify the models A–E, substantially reducing the overall computational cost.

### 4.2. Runtime and Quantization Analysis on Raspberry Pi Zero 2 W

Before evaluating deployment on constrained microcontrollers, the five selected models were analyzed for performance on the Raspberry Pi Zero 2 W. This step quantifies the impact of framework choice and quantization on real on-device performance, establishing the most efficient configuration for edge deployment.

#### 4.2.1. Framework Comparison: LiteRT vs. ONNX

**Figure 6:**
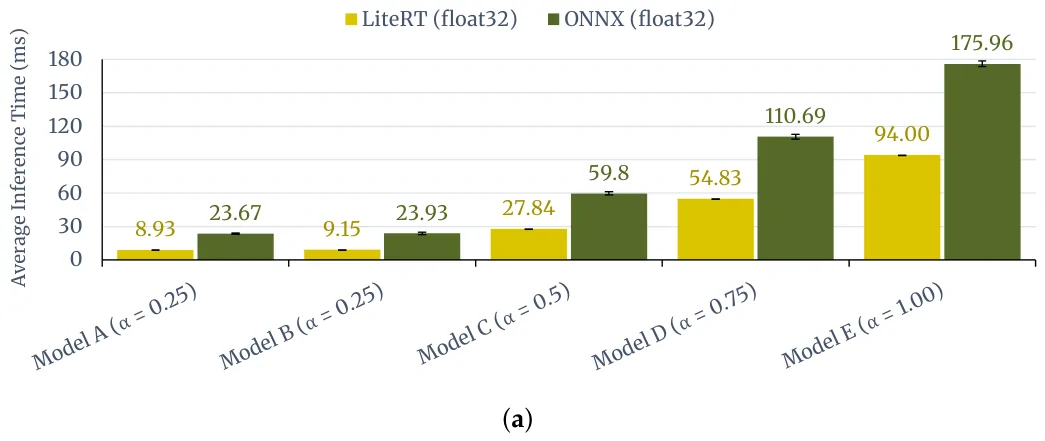

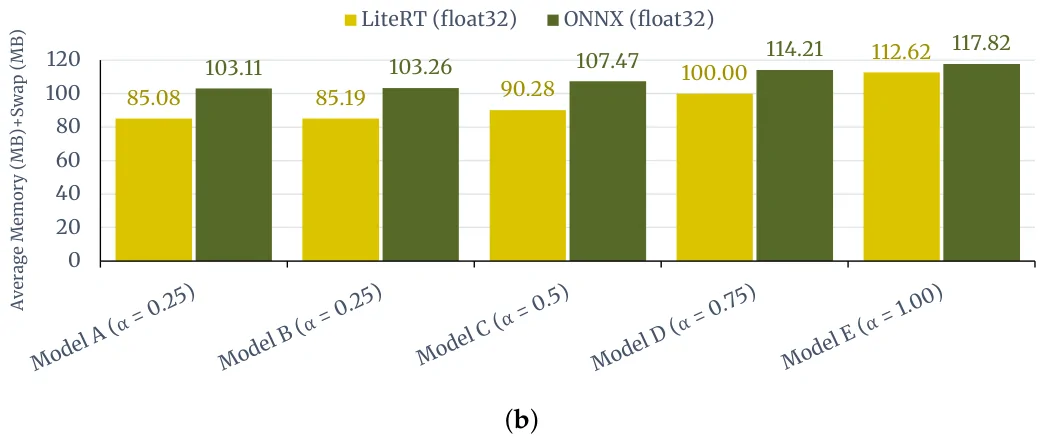
LiteRT vs. ONNX: (**a**) Inference time comparison; (**b**) Memory usage comparison.

To identify the most efficient inference framework for the resource-constrained Raspberry Pi Zero 2 W board, all five models (A through E) were benchmarked using both the LiteRT and ONNX frameworks with 32-bit floating-point precision (float32). As shown in Figure 6, LiteRT was faster than ONNX Runtime for all five models, by a factor of 2.65× for the lightest (8.93 ms against 23.67 ms) to 1.87× for the largest (94.00 ms against 175.96 ms). These differences reflect framework-specific choices in backend optimization and operator execution on the target ARM-based platform.

In addition to speed, LiteRT also showed lower memory consumption and the analysis reveals that models required less memory than their ONNX counterparts. For instance, Model A on LiteRT used 85.08 MB of memory, while the same model on ONNX used 103.11 MB. Similarly, Model E consumed 112.62 MB with LiteRT and 117.82 MB with ONNX, though the advantage was present across all models. The reported memory figures reflect the RSS (Resident Set Size) memory of the entire inference process, including the operating system overhead, Python runtime, and LiteRT framework libraries. Given the lower inference latency and reduced memory footprint, LiteRT was selected as the reference framework for deployment on the Raspberry Pi Zero 2 W and for all subsequent quantization experiments.

#### 4.2.2. Performance Gains from int8 Quantization

After selecting the LiteRT runtime, post-training mixed-precision quantization was applied to further optimize the models. The models were successfully optimized; the resulting model’s graph shows an initial QUANTIZE operation that converts the float32 input tensor to int8. Subsequently, all core computational layers operate entirely using int8 precision. A final DEQUANTIZE operation at the end of the graph converts the integer-based result back to a float32 output (for compatibility with floating-point post-processing, logging, and monitoring interfaces), consistent with our optimization strategy. In our case, this approach is equivalent to full integer quantization of the model, while defining QUANTIZE and DEQUANTIZE functions to maintain a float32 public interface. The number of parameters and model sizes before and after quantization across all models (A–E ) are shown in Table 7. The Keras column counts only the learned parameters, whereas the LiteRT columns report the total number of tensor elements allocated by the interpreter at inference time, which additionally includes the input/output tensors and the intermediate activation buffers of the graph, hence the “(+overhead)” notation. The quantized models show a constant increase in the number of elements over their float32 counterparts, corresponding exactly to the QUANTIZE operator’s int8 copy of the 128×128×3 input tensor and the 3-element float32 output of the final DEQUANTIZE operator described above. The learned weights themselves are preserved by quantization; the reduction in model size therefore stems from storing each weight as an 8-bit integer instead of a 32-bit float. The reduction ranges from 2.83× to 3.68× across models. It does not reach 4× because the .tflite container stores graph metadata and quantization parameters alongside the weights.

**Figure 7:**
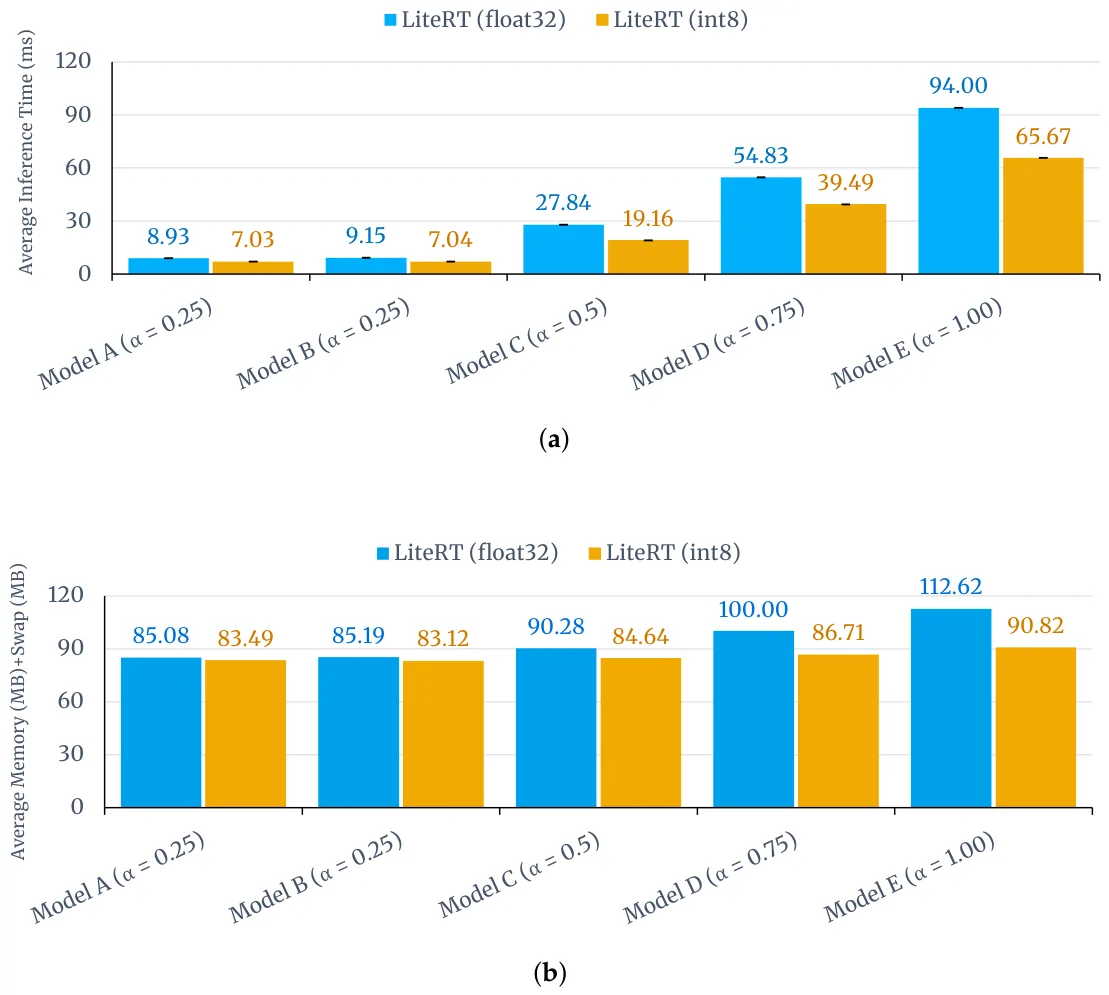
LiteRT vs. LiteRT-Quant: (**a**) Inference time comparison; (**b**) Memory usage comparison.

This step yielded performance improvements in both speed and memory usage, as detailed in Table 8 and illustrated in Figure 7. Inference time was reduced across all quantized models. For example, the inference time for Model C decreased from 27.84 ms (float32) to 19.16 ms (int8) corresponding to a 31.2% reduction. Model E saw its inference time drop from 94.00 ms to 65.67 ms, corresponding to a speed-up of approximately 30.1%. Quantization also led to a reduction in total memory consumption. For Model C, the total memory footprint was reduced from 90.28 for the float32 version to 84.64 MB for the int8 version. For Model E, the memory requirement fell from 112.62 MB to 90.82 MB, a 19.4% reduction that is critical for devices with limited RAM.

The comparison shown in Table 8 across models A–E establishes that int8 quantization improves speed and memory efficiency. However, models A–E differ in α, Bs, Dr, and *N* simultaneously. To characterize how the quantization trade-off varies with model width alone, Table 9 repeats the float32-vs-int8 comparison with Bs, Dr, and *N* fixed at Model C’s values and α varied across its four values.

Model C serves as the reference configuration because it represents the mid-range operating point at α = 0.5 and lies near the knee of the Pareto front. Since Table 4 reports optimizer-selected configurations rather than a controlled sweep, entries sharing a value of α across the two tables are not the same model.

Across all four values of α (Table 9), quantization reduces inference time by a similar proportion, between 21.4% and 31.2%, showing that the speed gain from int8 quantization does not depend strongly on model width. Memory savings, in contrast, grow with α: quantization reduces memory usage by only 2.7% at α=0.25 but by 18.6% at α=1.0, since larger models have more float32 weights to compress into int8. The accuracy cost of quantization stays small throughout, with F1 score dropping by no more than 0.0073 at any α, and does not increase systematically with model width.

#### 4.2.3. Evaluating the Accuracy–Quantization Trade-Off

**Figure 8:**
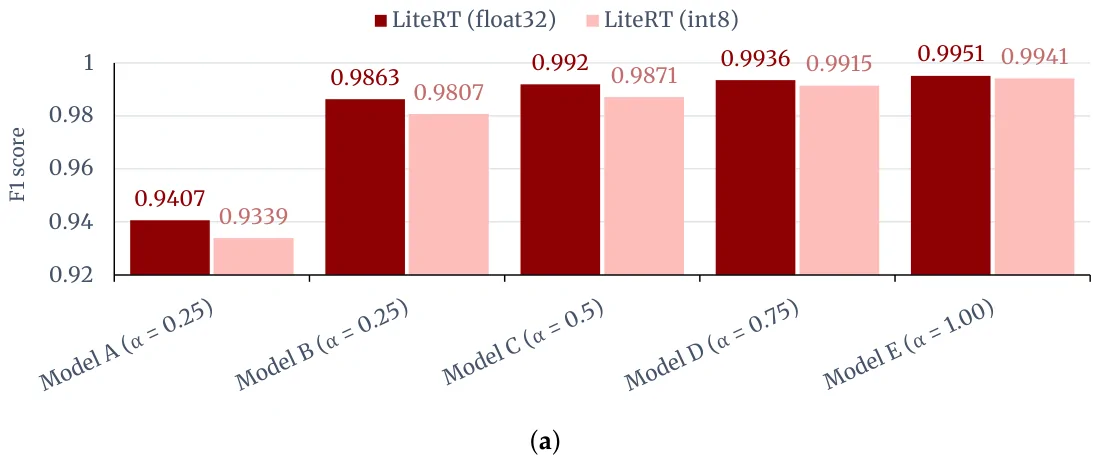

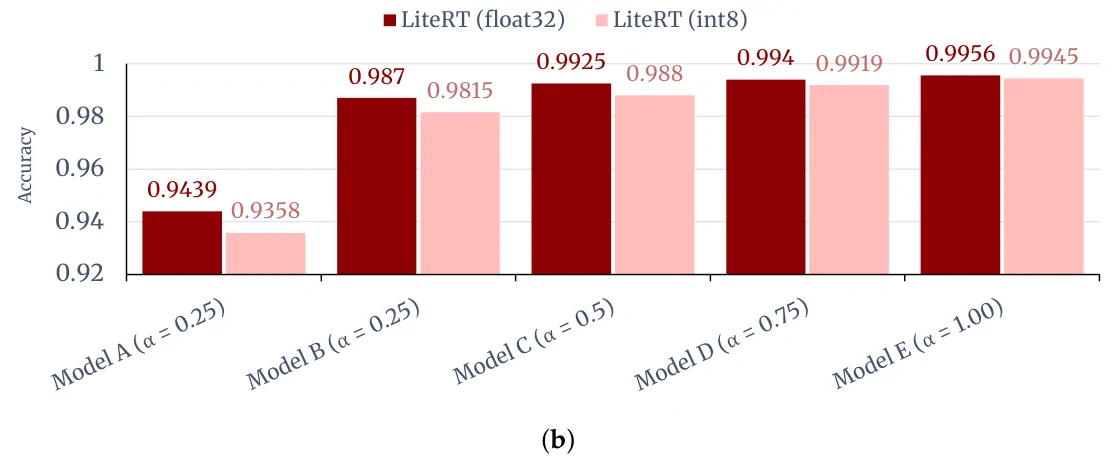
Comparison of performance degradation for LiteRT and LiteRT-Quant: (**a**) F1 score degradation; (**b**) Accuracy degradation.

While quantization enhances performance, it can potentially degrade model accuracy. This trade-off was evaluated by measuring the change in Accuracy and F1 score. To determine whether quantization systematically altered classification outcomes, we conducted 30 independent post-training quantization runs for each of Models A–E. In each run, a different representative dataset was randomly sampled from the training set. We then applied McNemar’s test to the paired predictions generated by the float32 and int8 models on the same test samples. The test is based on the discordant counts *b* and *c*, where *b* denotes the number of samples correctly classified only by the float32 model and *c* denotes the number correctly classified only by the int8 model. Under the null hypothesis that quantization does not systematically alter classification outcomes, *b* and *c* are expected to be similarly distributed. As shown in Table 10, the mean value of *b* is greater than that of *c* for all five models, indicating a consistent tendency for quantization to convert some correct predictions into incorrect ones. Moreover, the total discordant count, b+c, decreases from Model A to Model E. Thus, disagreement between the float32 and int8 models becomes less frequent as the capacity of the selected model configurations increases. Accordingly, Table 11 shows that the paired difference is statistically significant at p<0.05 in all 30 runs for Models A–D and in 25 of the 30 runs for Model E, with the corresponding decrease in F1-score remains below 0.007 for every model. For Model E, the average int8 accuracy remained at 0.9945 ± 0.0001, compared with 0.9956 for the float32 model, while the F1 score remained at 0.9941 ± 0.0001, compared with 0.9951. For Model A, the lightest configuration, the average int8 F1 score was 0.9339 ± 0.0007, compared with 0.9407 for float32 (Figure 8). These findings indicate that quantization introduces a small, reproducible reduction in predictive performance, with the magnitude of the effect becoming smaller for the higher-capacity configurations.

**Figure 9:**
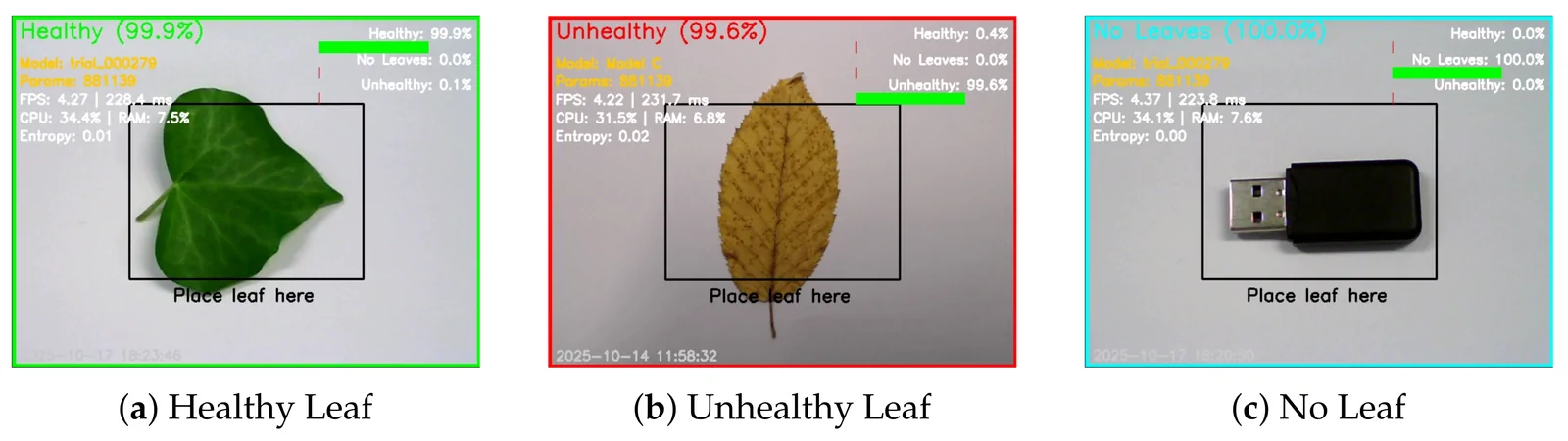
Example of real-time on-device inference showing predicted output with corresponding confidence and runtime metrics such as latency and resource usage. (**a**) is a healthy leaf (99.9%); (**b**) is an unhealthy leaf (99.6%); (**c**) is a no leaf (100.0%).

Finally, Figure 9 shows on-device inference during a demonstration in which locally collected leaves were tested on the Raspberry Pi Zero 2 W. Images were acquired with a Logitech C270 USB webcam (1280 × 720) mounted on a tripod in top-down orientation approximately 25 cm above the sample, under ambient indoor illumination. A fixed central region of interest, indicated by the on-screen guide, was cropped from each frame, rescaled to 128 × 128 pixels, and passed to the deployed model. The latency and frame rate overlaid in Figure 9 refer to the complete capture-to-display pipeline and therefore exceed the inference-only latencies reported in Table 8. The demonstration showed the end-to-end operation on previously unseen, real-world samples, providing a qualitative proof of concept beyond the PlantVillage benchmark.

### 4.3. Deployment and Execution Analysis on STM32 Microcontroller

Deploying deep learning models on devices with stringent, limited computational resources, such as an STM32 microcontroller (STM32H743ZI), poses significant challenges due to memory constraints, computational throughput, toolchain compatibility, and real-time responsiveness, all of which directly affect performance and overall deployment feasibility.

#### 4.3.1. Models Deployment Feasibility

The model obtained by the training phase, or even after the post-training quantization phase, still requires additional processing before it can be executed on an MCU. At this stage, dedicated toolchains, typically provided by silicon vendors, such as STM X-CUBE-AI and stedgeai-core, are used to craft the computing environment. The resulting output, i.e., a validation firmware, is C code that embeds a set of evaluation features, exposed through APIs, allowing developers to profile neural network execution on the MCU. These profiling capabilities include overall and per-layer performance metrics such as latency, RAM usage, multiply–accumulate operations, and CPU cycles. Since the firmware expects the model to be embedded directly into the C source, it becomes part of the compiled binary. The conversion from the original model to C code is handled by the stedgeai-core toolchain, which supports multiple input formats (e.g., Keras, TFLite, ONNX) and maps model operations (e.g., convolutional layers, dense layers, dropout layers) to the corresponding silicon-specific implementations. If the conversion of even a single operation fails, the entire model conversion process is aborted, and the model is declared incompatible with the target MCU. Moreover, the input model format can influence the success of this process, as certain formats may express operations or layer configurations in ways that are less compatible with the target toolchain or its supported operator set. Subsequently, during compilation and linking, the toolchain performs static verification to ensure that the generated firmware fits within the RAM and flash memory available on the MCU. If the memory requirements exceed the device’s capacity, the toolchain reports the overflow amount, indicating by how much the firmware exceeds the target memory limits. As a consequence, the model deployment fails.

**Figure 10:**
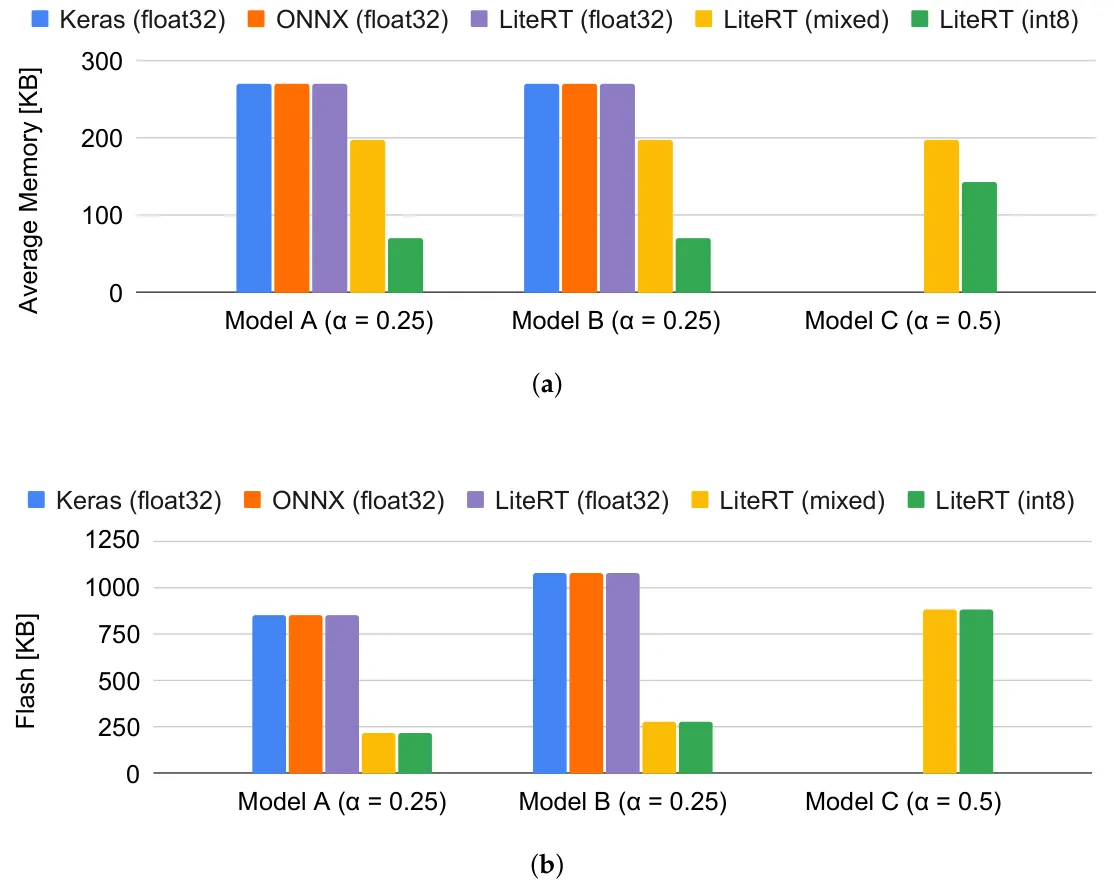
Comparison among different configurations and models: (**a**) Memory usage comparison across different configurations; (**b**) Flash memory footprint comparison.

Considering the five models identified and selected in Section 4.1, each model undergoes the processing and verification procedures described above to enable deployment on the target STM32H743ZI MCU, which provides 1 MB of RAM and 2 MB of flash memory. The outcomes of the model conversion, compatibility verification, and static resource analysis are summarized in Table 12. The table reports, for each model and configuration (such as format, quantization, and precision), the actual deployability outcome (success or failure reason) along with a few metrics such as the total number of parameters (including any additional quantization parameters), the flash and RAM footprint, and the number of multiply–accumulate (MACC) operations required to perform a single inference (see Figure 10).

This analysis reveals that models A and B can be successfully deployed on the MCU across multiple quantization–precision–format configurations without exceeding the available on-chip memory (1 MB of RAM and 2 MB of flash). Considering the float32 Keras model as baseline configuration, the RAM usage reaches 270,432 bytes, corresponding to about 26% of the available RAM, while the flash footprint amounts to 850,716 bytes (42%) and 1,076,396 bytes (53%) for models A and B, respectively. When using the fully quantized int8 TFLite version, the RAM footprint decreases to 71,232 bytes (7%), resulting in a 74% memory saving that can be used for additional processing tasks. Similarly, the flash footprint drops to 220,924 bytes (11%) and 277,996 bytes (14%) for models A and B, respectively, corresponding to a 74% reduction. All other evaluated configurations exhibit memory usage within these two bounds, confirming that the deployability of models A and B remains stable across different quantization, precision, and format combinations. In contrast, model C can be deployed on the MCU only when a compression technique, such as quantization, is applied to reduce its memory footprint, together with the use of the TFLite runtime. Under these conditions, the fully quantized int8 model achieves a minimum footprint of 142,464 bytes of RAM (14%) and 881,448 bytes of flash memory (43%). The RAM footprint increases to 196,608 bytes (19%) when using a mixed int8 quantization, as additional RAM is required for the storage and for the quantization and dequantization operations on the input and output tensors. All other configurations exceed the available RAM or flash memory, resulting in conversion and deployment failures. Finally, all configurations for models D and E fail the deployment process because their memory requirements exceed the target MCU’s available in both RAM and flash. However, it is worth noting that configurations using TFLite with quantization exceed only the flash memory limit for both models D and E, while still fitting within the available RAM.

#### 4.3.2. Runtime Performance Analysis

The analysis conducted in the previous section identified 12 valid configurations (5 for model A, 5 for model B, and 2 for model C) that can be safely deployed on the STM32H743ZI MCU. However, having deployable models does not necessarily guarantee that their execution performance meets real-time or runtime constraints; these constraints must be properly evaluated. For this reason, a runtime analysis was performed using the stedgeai-core toolkit to profile each configuration. 10 on-device inferences per configuration were run, after a single warm-up inference to allocate tensors. The input data consisted of randomly generated images with the correct dimensions for the evaluated networks, i.e., 128×128×3.

**Figure 11:**
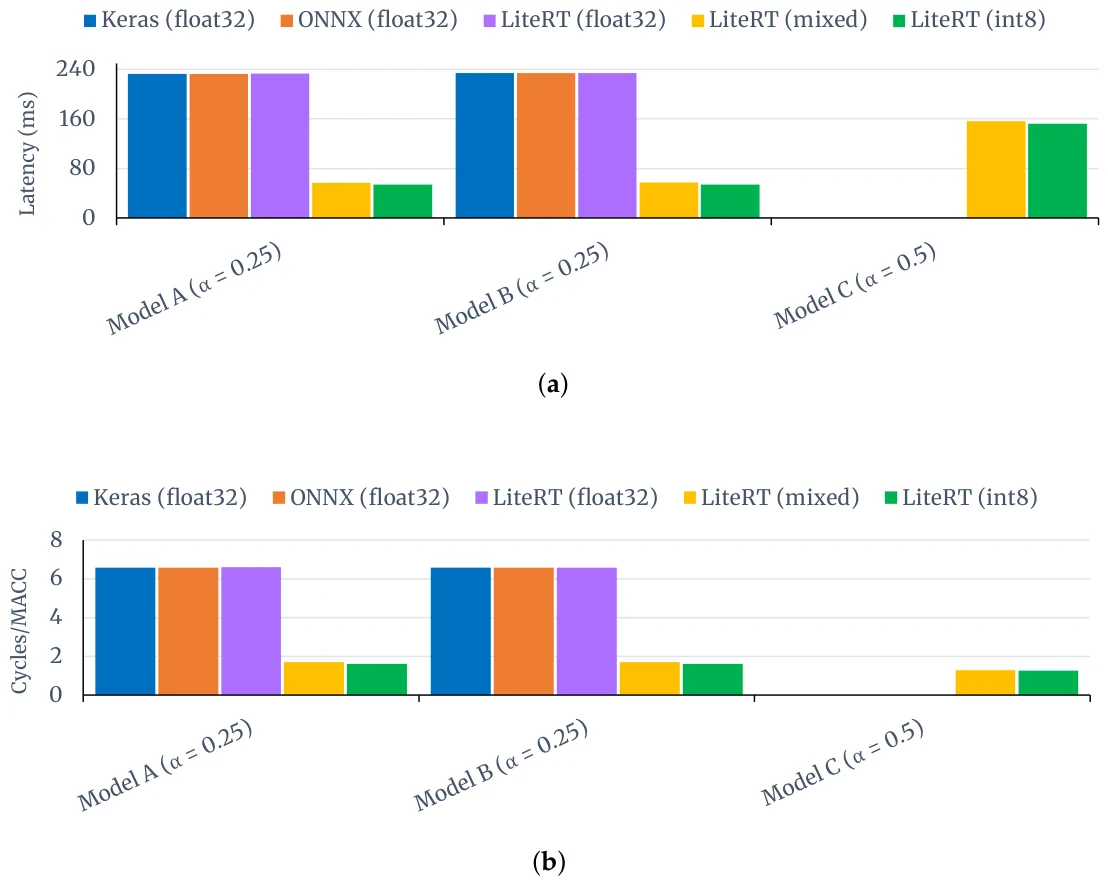
Runtime performance comparison among different configurations and models: (**a**) latency comparison; (**b**) cycles-per-MACC comparison.

The results are summarized in Table 13, and Figure 11 providing a detailed overview of the runtime behavior of each deployable configuration.

The profiling results show differences in performance across models and configurations, particularly in terms of latency, throughput (FPS), and resource utilization. For models A and B, which are deployable at both precisions, quantization reduces latency by 4.3×, from 232.52 ± 0.017 ms (for the float32 Keras model) to 53.928 ± 0.008 ms (for the fully quantized int8 TFLite version) in model A, and from 233.903 ± 0.011 ms to 54.194 ± 0.004 ms in model B. As a consequence, given that an embedded computer vision application is delivered, the FPS throughput increases from approximately 4.3 FPS to 18.5 FPS for model A and from 4.3 FPS to 18.4 FPS for model B. Regarding model C, only quantized configurations can be successfully executed on the MCU, as non-quantized versions exceed the available memory limits and fail the deployment process, as discussed in Section 4.3.1. The fully quantized int8 TFLite version has an inference latency of 152.553 ± 0.020 ms, while the mixed-precision configuration reaches 156.286 ± 0.025 ms. These values correspond to an inference throughput of approximately 6.6 FPS and 6.4 FPS, respectively, still within the range of real-time model inference for lightweight embedded applications.

In addition to latency and throughput, CPU cycles and the corresponding cycles-per-MACC ratio were analyzed, providing further insight into computational efficiency; for both quantities, lower values indicate more efficient use of processing resources. Model A shows a reduction from 93.09 Mcycles (million CPU cycles) with an efficiency of 6.58 cycles/MACC in the float32 Keras configuration to 21.57 Mcycles (1.62 cycles/MACC) in the fully quantized int8 TFLite version. Similar behaviors are observed for models B and C, where lower CPU cycle counts and cycles-per-MACC ratios indicate more efficient use of processing resources. These findings provide an initial indication of potential energy-efficiency gains, since reduced cycle counts correspond to shorter CPU active times and, consequently, lower battery consumption during inference. This is an essential factor if the final device is expected to operate autonomously or far from a stable power source.

### 4.4. Discussion & Limitations

The results presented above demonstrate the technical feasibility of the proposed TinyMLOps pipeline across heterogeneous edge targets. Here, we discuss the broader implications and limitations of these findings, quantify the effect of increasing the output-class granularity in Section 4.4.1, and outline a roadmap toward field-ready deployment in Section 4.4.2.

This work was developed and evaluated under controlled laboratory conditions. The use of PlantVillage introduces a potential domain shift when moving to open-field conditions characterized by variable illumination, occlusions, and complex backgrounds. Consequently, the predictive performance measured under controlled benchmark conditions may not fully reflect model robustness in real agricultural environments.

The original 39 fine-grained classes were aggregated into three superclasses (i.e., healthy leaves, unhealthy leaves, and no leaves) to define a coarse-grained plant-health screening task. This formulation supports health-status assessment and background rejection, but not species- or disease-specific diagnosis. Therefore, the proposed system is suitable for preliminary screening and early triage, rather than treatment guidance. Section 4.4.1 quantifies the memory impact of increasing the output size from three up to 39 classes.

Finally, energy consumption was not directly measured; CPU-cycle reduction after quantization was instead used as an implementation-level indicator of improved execution efficiency. Direct energy profiling is included in our roadmap (Section 4.4.2).

#### 4.4.1. Class-Count Scalability Under a Fixed Architecture

To assess the memory impact of increasing the model classification granularity, we consider the current model architecture while keeping the MobileNetV1 backbone, hidden-layer width, input resolution, and quantization configuration unchanged.

Let *N* be the hyperparameter defining the number of units in the last hidden classification layer and *K* the number of output classes. The final dense layer contains N·K weights and *K* biases; therefore, its parameter count is:

(3) $$P_{\mathrm{out}}\left(K\right)=N\cdot K+K=\left(N+1\right)\cdot K.$$

Increasing the output from three superclasses to the original 39 classes therefore adds:

(4) $$\Delta P=P_{\mathrm{out}}\left(39\right)-P_{\mathrm{out}}\left(3\right)=36\cdot \left(N+1\right)$$

parameters.

Considering the five selected models reported in Table 4, Table 14 reports the current parameter count and the projected count obtained by increasing the output layer from 3 to 39 classes according to Equation (4).

To obtain deployment-level measurements, Models A-C were converted from 3 to 39 output classes by widening the output layer only, with randomly initialized head weights, and were used exclusively for static memory analysis; no training or predictive evaluation was performed on them. Only the full-int8 LiteRT configuration was considered, as it is the most memory-efficient configuration evaluated and the most relevant deployment option for the resource-constrained MCU; Model C, in particular, fits the target hardware only under a quantized LiteRT configuration. Models D and E were excluded because their full-int8 configurations already exceed the MCU flash budget with three classes.

As shown in Table 15, all three 39-output architectural variants pass the static deployment checks for the STM32H743ZI, and the measured growth follows the analytical trend of Equation (4). The measured flash growth is ΔFlash=36N+144 B for all three models, matching the storage of the added tensors under full-int8 quantization: 36N weights at one byte each, plus 36 biases stored at int32 precision (144 B). No residual serialization overhead is observed, so the flash cost of additional classes is linear in *N* and independent of the backbone width. The toolchain-reported increase, $\Delta P_{\mathrm{tool}}=36N-3$, falls 39 parameters below the analytical 36(N+1) because the zero-initialized bias of the untrained widened head is not serialized, though it is still allocated in flash. In all cases, these increments are small relative to the available 2 MB flash budget.

No increase in RAM is reported for any model. Increasing the class count mainly adds final-layer weights and biases, which are stored in flash. Peak RAM usage remains determined by the larger intermediate activation tensors of the MobileNetV1 backbone. The expansion of the output tensor from 3 to 39 int8 elements is too small to alter the peak, aligned activation-memory allocation reported by the toolchain.

#### 4.4.2. Roadmap Towards Field Deployment

Closing the gap between the controlled, laboratory-acquired PlantVillage benchmark and real open-field conditions is a prerequisite for operational deployment. This section outlines a staged roadmap that builds directly on lifecycle infrastructure and methodology already established in this work.

**Field-oriented augmentation and negative-sample collection.** As an initial step, the augmentation strategy described in Section 3.1.1 will be extended to better approximate open-field conditions by compositing leaf images onto natural backgrounds, simulating partial occlusion, and introducing broader variations in illumination, shadows, blur, and viewpoint. In parallel, representative field-negative images (e.g., soil, weeds, sky, equipment, and human hands) will be collected opportunistically to strengthen the no leaves class without requiring disease-specific annotation. Together, these measures are expected to improve background rejection and reduce the domain gap between laboratory and field imagery.

**Field-data acquisition and validation.** A representative field dataset will be collected using the intended camera configuration and preprocessing pipeline, covering variations in location, illumination, viewpoint, occlusion, growth stage, and natural background. The PlantVillage-trained models will first be evaluated directly on these images to quantify the laboratory-to-field domain shift and will then be fine-tuned using field-acquired samples. Evaluation partitions will be separated by location, plant, date, or acquisition session rather than by random image-level splitting. Performance will be assessed using standard classification and system-level metrics under field conditions. Updated models will also be re-evaluated for deployment feasibility on the target devices before redeployment.

**Toward fine-grained diagnosis.** The current three-superclass model acts as a lightweight first-stage screening mechanism, and the same architecture can be specialized without changing the deployment pipeline. For instance, the three-class output can be fine-tuned for a single species, such as tomato, to improve the assessment of species-specific health conditions. Extending the pipeline to fine-grained disease classification while preserving MCU feasibility is therefore a core direction for future work. Any resulting change to the model or input resolution would in turn be subject to the same deployment verification procedure applied in Section 4.3.1.

**System-level field validation.** Field validation will include direct power measurements and RGB-camera integration on both platforms, complementing the CPU-cycle results in Section 4.3.2 with system-level evaluation under real-world conditions. Complementary measurements from temperature, humidity, and soil-moisture sensors will also be incorporated to improve monitoring reliability, enabling the vision-sensing node to be evaluated jointly with the broader environmental context of the deployment site.

## 5. Conclusions

This paper presented a TinyMLOps pipeline for operationalizing plant disease classification models on heterogeneous low-power edge devices. The proposed workflow integrates data preparation, model adaptation, multi-objective hyperparameter optimization, deployment-oriented conversion, profiling, on-device inference, and monitoring into a unified lifecycle.

The experimental results show that combining a MobileNet-based architecture with multi-objective optimization and post-training quantization yielded configurations deployable on both target platforms. Relative to the LiteRT float32 models, the int8 models were 2.83–3.67× smaller on disk, and inference latency on the Raspberry Pi Zero 2 W fell by 21.3–31.2%, at a cost in F1-score of between 0.0010 and 0.0068. LiteRT also gave lower latency and memory usage than ONNX Runtime at float32 for all five models, by factors of 2.65× (Model A) to 1.87× (Model E) in latency. On the STM32H743ZI, Models A and B deployed successfully under all five format–precision combinations tested, Model C deployed only as a quantized LiteRT model, and Models D and E failed static verification in every configuration; for Models A and B, the fully quantized int8 counterparts are 4.3× faster and 3.85× smaller in MCU flash footprints than the float32 variants.

Overall, the results demonstrate the feasibility of a lifecycle-oriented TinyMLOps approach for precision agriculture vision tasks, in which accuracy, memory footprint, inference latency, and deployment compatibility must be optimized jointly. The modular structure of the pipeline supports transferability across edge targets and provides a foundation for future extensions toward field validation, direct energy profiling, and more fine-grained plant disease classification while preserving edge deployability. Future work will focus on the field-deployment roadmap outlined in Section 4.4.2, integrating the proposed pipeline with an RGB camera module and complementary environmental sensors as a complete edge vision-sensing node for greenhouse and open-field monitoring.

## Figures and Tables

**Table 1:** Summary of related works. NR: Not Reported.

Paper	ML Model	Metric	Target Environment	Dataset	Preprocessing	Classes	Framework	Val Domain
Aqasizade et al. [17]	MobileNet	Classification Performance, Model Size, Inference Speed, Memory Usage	Raspberry Pi Zero 2 W	Plant Village	Augmentation	39 Classes, 3 Superclasses	LiteRT	Plant Disease
Antonini et al. [5]	Isolation Forest	Deployment, Inference, and Loading Latency, Memory Utilization	Raspberry Pi 4 B, MCU (ESP32)	NASA Bearing, Vibrational Recordings	Feature Extraction, Feature Engineering	2 Classes	Pure MicroPython	Industrial wastewater management plant
Khan et al. [11]	MobileNetV3-Small	Accuracy, F1 score	Edge devices	PlantVillage	*NR*	38 Classes	PyTorch, ONNX	Plant Disease
Leroux et al. [23]	Deep Neural Network	*NR*	Resource-Constrained Edge Devices	*NR*	*NR*	*NR*	*NR*	TinyMLOps Challenges
Peltonen et al. [24]	Logistic Regression, Linear SVM, Decision Tree, Random Forest, XGBoost, Adaboost, and Custom Ensemble	accuracy and F1score, CPU and Memory Usage, Inference Time	Raspberry Pi 4 B	Breakdown of Air Pressure Systems in Trucks	Feature Removal and Imputation of Missing Values	2 Classes	LinkEdge	Predictive Maintenance
Min et al. [25]	SensiX++ with 10 Different Vision and Acoustics Models	Throughput, Latency	Jetson AGX, Coral TPU	*NR*	Data Transformations, Feature Extraction	*NR*	SensiX++	MLOps, Multi-Tenant Model Serving
Huang et al. [26]	RIOT-ML	RAM, Flash Memory Usage, Computational Latency	Resource-Constraint IoT Devices and Microcontrollers	*NR*	Quantization	*NR*	RIOT-ML Toolkit	Local and Remote IoT Boards
Larian et al. [18]	CNN-LSTM	Response Time, Network Traffic, Throughput, Edge Energy Consumption, Cloud Energy Consumption, Accuracy, F1 score	Raspberry Pi 2B, Raspberry Pi 3B	MHEALTH	Conversion To a Standardized Format, Normalization, Categorization	12 Classes	InTec, LiteRT	Human Motion Detection in Smart Homes
Jenhani et al. [19]	MobileNetV2	Latency, Round-Trip Time (RTT), Packet Count	MCU (ESP32)	Pretrained on ImageNet	Quantization	*NR*	TensorFlow Lite (TFLite) and TensorFlow Lite Micro (TFLM)	Wireless Protocol Benchmarking for Distributed TinyML Inference
Hayajneh et al. [20]	CNN-LSTM-TL	Classification Accuracy	Raspberry Pi 4 & MCU	MotionSense and UCI	Augmentation & PCA	MotionSense Dataset: 5 Classes, UCI HAR Dataset: 6 Classes.	TinyMLaaS	Cross-Dataset Human Activity Recognition
Karras et al. [27]	Suite of TinyML Algorithms	Clustering Speed, Compression Efficiency, Compression Speed, Resource Utilization, Adaptability Score	Raspberry Pi 4 B, Raspberry Pi Zero W	Raw Sensor Data	Augmentation	*NR*	Flask Server	Big Data in Large-Scale IoT Systems
Cerioli et al. [21]	MLP, GRU	Inference Time, Ram and Flash Usage	STM32H747XI -M7/M4, nRF52840, ESP32PICO-D4, Raspberry Pi 4	Synthetic Data	Quantization	2 Classes	NeuralCasting	Environmental Monitoring
Lê et al. [22]	GRU, CNN, RNN	Accuracy, Model Size, Stack Size, Code Memory, Latency	MCU (Arm Cortex-M0+ and M4)	Head Gesture, Activity Recognition	*NR*	4 Classes (for the head gesture dataset); Activity Recognition Dataset is *NR*	LiteRT Quant, NNoM, Custom C	TinyMLOps
Katib et al. [28]	GLMS-BiLSTM	Accuracy, Precision, Recall, F-score, G-Measure	Microcontroller	Benchmark Dataset	Feature Selection, Data Normalization, and Missing Value Imputation	10 Classes	DLTML-RTADPM	IoT Security
Flores et al. [29]	A Multilayer Perceptron Neural Network	Mean Absolute Percent Error (MAPE)	MCU (ESP32)	Manually Generated by Collecting Data from Four Vehicles	Data Engineering & Feature Engineering	Quantization	LiteRT_Quant	Internet of Intelligent Vehicles
Arratia et al. [16]	TinyML CNN	Accuracy, Inference Cost, Energy Consumption, Latency	Edge Devices	ephemeral stream images, real-world field data)	Resizing, Expansion, Transformations	2 Classes	TinyML Toolchains	Streamflow Detection
Sakthipriya et al. [9]	GEN-CNN	Accuracy, Convergence Speed	Cloud / GPU servers	Soil and Crop Images	Feature Extraction, GA-based architecture optimization	multi-class	CNN-based, GA-driven optimization	Precision agriculture
Wadhwa et al. [8]	Hybrid DL ensemble	Accuracy, Precision, Recall, F1-score, ROC-AUC	Web, Mobile, IoT-compatible Inference Platforms	PlantVillage, PlantDoc, the Banana Leaf Disease, Coffee RoCoLe, IBean	Normalization	36 Classes	TensorFlow/Keras, CatBoost, SHAP	Plant disease detection
Sivakumar et al. [10]	Federated Ensemble Learning	MIoU, Accuracy, F1-score	Distributed Edge	Sugar Beet Crop, Carrot Crop, Sunflower Crop, Potatoes Crop, Maize Crop	Resizing, Normalization	2 Classes	Deep learning + Federated Learning frameworks	Precision agriculture
This Work	MobileNet	Classification Performance, Model Size, Inference Speed, Memory Usage	Raspberry Pi Zero 2 W, MCU	Plant Village	Augmentation, Quantization	39 Classes, 3 Superclasses	LiteRT, LiteRT_Quant, ONNX	Plant Disease

**Table 2:** Mapping of the initial PlantVillage dataset classes to superclasses.

Healthy Leaves	Unhealthy Leaves	No Leaves
Apple Healthy	Apple Apple scab	Background Without
Blueberry Healthy	Apple Black Rot	Leaves
Cherry Healthy	Apple Cedar Apple Rust	
Corn Healthy	Cherry Powdery Mildew	
Grape Healthy	Corn Cercospora Leaf Spot Gray Leaf Spot	
Peach Healthy	Corn Common Rust	
Pepper Bell Healthy	Corn Northern Leaf Blight	
Potato Healthy	Grape Black Rot	
Raspberry Healthy	Grape Esca (Black Measles)	
Soybean Healthy	Grape Leaf Blight (Isariopsis Leaf Spot)	
Strawberry Healthy	Orange Huanglongbing (Citrus Greening)	
Tomato Healthy	Peach Bacterial Spot	
	Pepper, bell Bacterial Spot	
	Potato Early Blight	
	Potato Late Blight	
	Squash Powdery Mildew	
	Strawberry Leaf Scorch	
	Tomato Bacterial Spot	
	Tomato Early Blight	
	Tomato Late Blight	
	Tomato Leaf Mold	
	Tomato Septoria Leaf Spot	
	Tomato Spider Mites, Two-spotted Spider Mite	
	Tomato Target Spot	
	Tomato Mosaic Virus	
	Tomato Yellow Leaf Curl Virus	
12 Classes (16,468)	26 Classes (43,875)	1 Class (1143)

**Table 3:** Tunable hyperparameters used in the optimization process.

Hyperparameter	Description	Possible Values
α	Width multiplier	{ 0.25, 0.5, 0.75, 1.0 }
Dr	Dropout rate	{ 0, 0.1, 0.3, 0.5, 0.7, 0.9 }
Bs	Training batch size	{ 4, 8, 16, 32 }
*N*	Last layer size	8≤N≤512, N∈Z

**Table 4:** Comparison of Keras models A–E (four non-dominated Pareto-front models and one dominated reference model). Performance computed during the hyperparameter optimization phase.

Models	Complexity	Status	Hyperparameters				Total Parameters	F1 Score
			** α **	** Bs **	** Dr **	** N **		
A	Lightest	Non-dominated	0.25	16	0.5	9	220.88 K	0.965
B	Light	Non-dominated	0.25	32	0.3	226	277.30 K	0.990
C	Mid-range	Non-dominated	0.5	16	0.1	100	881.13 K	0.994
D	Heavy	Non-dominated	0.75	16	0	359	2.11 M	0.996
E	Heaviest	Dominated	1	32	0.1	479	3.72 M	0.996

**Table 5:** Parameter–efficiency comparison of our five selected Keras models against PlantVillage baselines.

Work	Architecture	Task Granularity	Model Size	F1 Score
Proposed Model A	MobileNetV1, α=0.25	3 Superclasses	0.221 M	0.965
Proposed Model B	MobileNetV1, α=0.25	3 Superclasses	0.277 M	0.990
Proposed Model C	MobileNetV1, α=0.5	3 Superclasses	0.881 M	0.994
Proposed Model D	MobileNetV1, α=0.75	3 Superclasses	2.11 M	0.996
Proposed Model E	MobileNetV1, α=1.0	3 Superclasses	3.72 M	0.996
Khan et al. (2023) [11]	MobileNetV3-Small	Fine-grained	0.9 M	0.995
Schuler et al. (2022) [15]	Modified Inception V3	Fine-grained	5 M	0.992
Mohanty et al. (2016) [12]	GoogLeNet	Fine-grained	5 M	0.983
Mohanty et al. (2016) [12]	AlexNet	Fine-grained	60 M	0.978
Toda et al. (2019) [13]	Inception V3	Fine-grained	5 M	0.972
Geetharamani et al. (2019) [14]	9-layer CNN	Fine-grained	0.2 M	0.981

**Table 6:** Estimated computational cost of the grid search for discovering models A–E, under sequential single-threaded execution and fixed traversal order.

Model	Config (α, Dr, Bs, N)	Trials	Estimated Minutes	Estimated Days
A	0.25, 0.5, 16, 9	7072	97,594	67.8
B	0.25, 0.3, 32, 226	5774	79,681	55.3
C	0.5, 0.1, 16, 100	15,243	210,978	146.5
D	0.75, 0.0, 16, 359	25,602	361,724	251.2
E	1.0, 0.1, 32, 479	40,367	657,695	456.7

**Table 7:** Comparison of models A–E converted to the LiteRT framework at float32 and int8 precision. The Keras column counts only the learned parameters, while the LiteRT columns count all tensor elements allocated at inference time (weights plus input/output and intermediate activation buffers, and, for int8, the boundary quantize/dequantize operators).

Models	Complexity	Total Parameters (+Overhead)			Model Size (KB)		
		Keras	float32	int8	Keras	float32	int8
A	Lightest	220,887	673,752	722,907	1198.00	847.01	299.13
B	Light	277,307	730,389	779,544	1859.21	1067.70	360.28
C	Mid-range	881,139	1,737,791	1,786,946	4162.02	3394.36	1010.77
D	Heavy	2,110,127	3,370,734	3,419,889	10,724.85	8163.03	2281.91
E	Heaviest	3,721,279	5,385,702	5,434,857	18,700.21	14,424.53	3922.59

**Table 8:** Comparison of average inference latency and average memory consumption between LiteRT float32 and post-training quantized LiteRT int8 models deployed on the Raspberry Pi Zero 2 W. Values are reported as mean ± standard deviation over 1000 runs for the five selected models (A–E).

Models	Inf. Time (ms) float32	Inf. Time (ms) int8	Memory + Swap (MB) float32	Memory + Swap (MB) int8
A	8.93±0.1963	7.03±0.0304	85.08±0.0278	83.49±0.0275
B	9.15±0.0453	7.04±0.0317	85.19±0.0279	83.12±0.0274
C	27.84±0.0931	19.16±0.0536	90.28±0.0278	84.64±0.0274
D	54.83±0.1113	39.49±0.0734	100.00±0.0278	86.71±0.0274
E	94.00±0.1692	65.67±0.0918	112.62±0.0277	90.82±0.0274

**Table 9:** The int8 quantization speed–accuracy trade-off across the width multiplier α, with all other hyperparameters frozen at Model C’s values (Bs=16, Dr=0.1, N=100) on the Raspberry Pi Zero 2 W. Values are reported as mean ± standard deviation over 1000 runs. The bold row (α=0.5) corresponds to Model C in Table 4.

Hyperparameters				Total Parameters	F1 Score float32	F1 Score int8	Inf. Time (ms) float32	Inf. Time (ms) int8	Memory + Swap (MB) float32	Memory + Swap (MB) int8
** α **	** Bs **	** Dr **	** N **							
0.25	16	0.1	100	244.55 K	0.9837	0.9764±0.0013	9.01±0.1978	7.08±0.1796	85.24±0.0276	82.95±0.0276
**0.5**	**16**	**0.1**	**100**	**881.13 K**	0.9920	0.9871±0.0001	27.84±0.0931	19.16±0.0536	90.28±0.0278	84.64±0.0274
0.75	16	0.1	100	1.91 M	0.9911	0.9847±0.0010	53.92±0.7078	39.37±0.6670	96.68±0.0272	86.51±0.0271
1.0	16	0.1	100	3.33 M	0.9952	0.9929±0.0007	94.60±1.7435	65.56±0.2604	109.34±0.0275	88.96±0.0272

**Table 10:** Paired classification outcomes of the float32 and int8 models on the fixed test set, reported as mean ± standard deviation over 30 quantization runs. Here, *a* and *d* denote agreement on correct and incorrect predictions, respectively, while *b* and *c* denote outcomes correct only for the float32 and int8 models, respectively.

Model	Both Correct (*a*)	Only float32 Correct (*b*)	Only int8 Correct (*c*)	Both Wrong (*d*)	Discordant (b+c)
A	13,958±11	327±11	204±6	645±6	531±13
B	14,811±5	126±5	43±3	154±3	169±6
C	14,921±1	99±1	31±1	83±1	130±1
D	14,985±2	58±2	27±1	64±1	85±2
E	15,033±1	34±1	18±1	50±1	52±1

**Table 11:** Predictive performance of the LiteRT float32 and int8 models on the fixed test set with deployment evaluation performed on the Raspberry Pi Zero 2 W. Values are reported as the mean ± standard deviation across 30 independent post-training quantization runs, each calibrated with a different random representative subset drawn from the training set. ΔF1 and ΔAccuracy are the corresponding decreases from float32 (mean ± std), and the last column gives the number of the 30 runs with a McNemar-significant float32–int8 difference at a significance level of *p* < 0.05.

Model	float32 F1 Score	int8 F1 Score	ΔF1 Score	float32 Accuracy	int8 Accuracy	ΔAccuracy	McNemar Significant
A	0.9407	0.9339±0.0007	0.0068±0.0007	0.9439	0.9358±0.0007	0.0081±0.0007	30/30
B	0.9863	0.9807±0.0004	0.0056±0.0004	0.9870	0.9815±0.0004	0.0055±0.0004	30/30
C	0.9920	0.9871±0.0001	0.0049±0.0001	0.9925	0.9880±0.0001	0.0045±0.0001	30/30
D	0.9936	0.9915±0.0001	0.0021±0.0001	0.9940	0.9919±0.0001	0.0021±0.0001	30/30
E	0.9951	0.9941±0.0001	0.0010±0.0001	0.9956	0.9945±0.0001	0.0011±0.0001	25/30

**Table 12:** Output of the model conversion, deployment verification, and static analysis on the STM32H743ZI MCU, showing deployability status, parameter count, flash and RAM footprint, and multiply–accumulate (MACC) operations for each model configuration (format, quantization, and precision).

Model	Input Format	Quantization	Precision	Parameters	Flash [B]	RAM [B]	MACC	Deployment Status
A	Keras	No	float32	215,415	850,716	270,432	14,146,573	Success
	LiteRT	Full	int8	212,679	220,924	71,232	13,323,268	Success
		Mixed	int8	212,679	220,924	196,608	13,421,578	Success
		No	float32	212,679	850,716	270,432	14,146,573	Success
	ONNX	No	float32	213,919	850,716	270,432	14,146,573	Success
B	Keras	No	float32	271,835	1,076,396	270,432	14,203,210	Success
	LiteRT	Full	int8	269,099	277,996	71,232	13,379,688	Success
		Mixed	int8	269,099	277,996	196,608	13,477,998	Success
		No	float32	269,099	1,076,396	270,432	14,203,210	Success
	ONNX	No	float32	270,339	1,076,396	270,432	14,203,210	Success
C	Keras	No	float32	870,195	3,458,892	540,864	50,360,196	RAM & Flash overflow
	LiteRT	No	float32	864,723	3,458,892	540,864	50,360,196	RAM & Flash overflow
		Full	int8	864,723	881,448	142,464	48,713,504	Success
		Mixed	int8	864,723	881,448	196,608	48,811,814	Success
	ONNX	No	float32	867,203	3,458,892	540,864	50,360,196	RAM & Flash overflow
D	Keras	No	float32	2,093,711	8,342,012	811,296	108,770,371	RAM & Flash overflow
	LiteRT	Full	int8	2,085,503	2,111,216	213,696	106,300,124	Flash overflow
		Mixed	int8	2,085,503	2,111,216	213,696	106,398,434	Flash overflow
		No	float32	2,085,503	8,342,012	811,296	108,770,371	RAM & Flash overflow
	ONNX	No	float32	2,089,223	8,342,012	811,296	108,770,371	RAM & Flash overflow
E	Keras	No	float32	3,699,391	14,753,788	1,081,728	189,190,219	RAM & Flash overflow
	LiteRT	Full	int8	3,688,447	3,722,728	284,928	185,896,556	Flash overflow
		Mixed	int8	3,688,447	3,722,728	284,928	185,994,866	Flash overflow
		No	float32	3,688,447	14,753,788	1,081,728	189,190,219	RAM & Flash overflow
	ONNX	No	float32	3,693,407	14,753,788	1,081,728	189,190,219	RAM & Flash overflow

**Table 13:** Runtime profiling results of deployable model configurations on the STM32H743ZI MCU.

Model	Framework	Quantization	Precision	Parameters	MACC	CPU Cycles	Cycles/MACC	Latency (Mean ± Std) [ms]
A	Keras	No	float32	215,415	14,146,573	93,088,129	6.580	232.52 ± 0.017
	LiteRT	Full	int8	212,679	13,323,268	21,571,202	1.619	53.928 ± 0.008
		Mixed	int8	212,679	13,421,578	22,852,505	1.703	57.131 ± 0.014
		No	float32	212,679	14,146,573	93,488,285	6.609	233.721 ± 0.021
	ONNX	No	float32	213,919	14,146,573	93,228,276	6.590	233.071 ± 0.011
B	Keras	No	float32	271,835	14,203,210	93,561,023	6.587	233.903 ± 0.011
	LiteRT	Full	int8	269,099	13,379,688	21,680,348	1.620	54.194 ± 0.004
		Mixed	int8	269,099	13,477,998	22,955,332	1.703	57.388 ± 0.014
		No	float32	269,099	14,203,210	93,591,315	6.589	233.978 ± 0.017
	ONNX	No	float32	270,339	14,203,210	93,557,548	6.587	233.894 ± 0.023
C	LiteRT	Full	int8	864,723	48,713,504	61,035,395	1.253	152.553 ± 0.02
		Mixed	int8	864,723	48,811,814	62,514,219	1.281	156.286 ± 0.025

**Table 14:** Architecture-level parameter growth when increasing the output layer from 3 to 39 classes, including output-layer and total model parameters.

Model	N	Total Parameters 3 Classes	$P_{\mathrm{out}}\left(3\right)$	ΔP	$P_{\mathrm{out}}\left(39\right)$	Total Parameters 39 Classes
A	9	220,887	30	360	390	221,247
B	226	277,307	681	8172	8853	285,479
C	100	881,139	303	3636	3939	884,775
D	359	2,110,127	1080	12,960	14,040	2,123,087
E	479	3,721,279	1440	17,280	18,720	3,738,559

**Table 15:** Measured deployment-level memory growth when increasing the output layer from 3 to 39 classes for the full-int8 LiteRT configurations of Models A–C. $\Delta P_{\mathrm{tool}}$, ΔFlash, and ΔRAM are computed relative to the corresponding three-class configuration.

Model	Classes	Parameters	$\Delta P_{\mathrm{tool}}$	Flash [B]	ΔFlash [B]	RAM [B]	ΔRAM [B]	Static Verification
A	3	212,679	–	220,924	–	71,232	–	Success
	39	213,000	321	221,392	468	71,232	0	Success
B	3	269,099	–	277,996	–	71,232	–	Success
	39	277,232	8133	286,276	8280	71,232	0	Success
C	3	864,723	–	881,448	–	142,464	–	Success
	39	868,320	3597	885,192	3744	142,464	0	Success

## Data Availability

The data used in this study are derived from the openly available GitHub repository available at https://github.com/FBK-OpenIoT/PlantVillage-AugNoLeaves, accessed on 1 July 2026.

## Abbreviations

The following abbreviations are used in this manuscript:

**Abbreviation**	**Description**
Op	Operation
int8	Integer8
LiteRT	Lite Runtime
ONNX	Open Neural Network Exchange
Quant	Quantized
InfTime	Inference Time
Mem	Memory
Avg	Average
GLMS	Gradient Least Mean Squares
BiLSTM	Bidirectional Long Short-Term Memory
